# JUND-DNAJB1-p21 axis drives fibroblast senescence to recruit CD4⁺ T cells in salivary gland pathogenesis of Sjögren’s disease

**DOI:** 10.1186/s12979-026-00590-8

**Published:** 2026-08-11

**Authors:** Yingliang Shi, Yi Guo, Hanlin Tu, Jiaqi Yue, Zhongyang Zou, Yuyan He, Jing Zhou, Xiang Li, Sangang He, Guoliang Sa

**Affiliations:** 1 State Key Laboratory of Oral & Maxillofacial Reconstruction and Regeneration, Key Laboratory of Oral Biomedicine Ministry of Education, Hubei Key Laboratory of Stomatology, School & Hospital of Stomatology, Wuhan University, Wuhan, 430079 China; 2 The Affiliated Stomatological Hospital of Chongqing Medical University, Chongqing, China; 3 Department of Stomatology, Huangshi Central Hospital, Affiliated Hospital of Hubei Polytechnic University, Huangshi, China; 4 Hubei Key Laboratory of Kidney Disease Pathogenesis and Intervention, Huangshi, 435000 China; 5 Department of Oral and Maxillofacial surgery, School and Hospital of Stomatology, Wuhan University, Wuhan, 430079 China

**Keywords:** Primary Sjögren's disease, Cellular senescence, Senolytics, Salivary gland fibroblasts, DNAJB1, JUND

## Abstract

**Background:**

Advanced age is a recognized risk factor for primary Sjögren’s disease (pSjD), suggesting a role for aging-related mechanisms in its pathogenesis. However, whether cellular senescence directly drives autoimmune inflammation and glandular dysfunction remains unclear. Furthermore, the specific role of senescent fibroblasts within the pSjD microenvironment has not been defined. This study investigates the mechanisms underlying fibroblast senescence and its therapeutic potential in pSjD.

**Methods:**

The senescence landscape of salivary glands was assessed by immunohistochemistry, multiplex immunofluorescence, and single-cell RNA sequencing, coupled with computational trajectory and gene regulatory network inference. In vitro, primary salivary gland fibroblasts (SGFs) were treated with interferon-gamma (IFN-γ). Senescence-associated β-galactosidase staining, reactive oxygen species detection, siRNA knockdown, co-immunoprecipitation, dual-luciferase reporter, and transwell migration assays were conducted to elucidate the underlying mechanisms. In vivo, a non-obese diabetic (NOD) mouse model was utilized to evaluate the efficacy of a senolytic combination (dasatinib and quercetin) on salivary flow rates and lymphocytic infiltration.

**Results:**

Integrative analysis of human clinical samples and scRNA-seq revealed a significant accumulation of senescent fibroblasts in pSjD salivary glands. Pharmacological clearance of senescent cells in NOD mice significantly attenuated lymphocytic infiltration and restored salivary function, supporting a pathogenic contribution of fibroblast senescence. Subsequent computational trajectories and in vitro validations identified a senescence-enriched DNAJB1^high^ fibroblast subpopulation. Mechanistically, IFN-γ upregulated DNAJB1 transcription via JUND, which was in turn associated with p21-mediated senescence and enhanced CD4⁺ T cell recruitment. Furthermore, in vivo evaluations confirmed that the senolytic intervention effectively suppressed the glandular JUND-DNAJB1-p21 axis, corroborating the mechanistic findings.

**Conclusions:**

These findings indicate that the JUND-DNAJB1-p21 axis contributes to fibroblast senescence and autoimmune pathogenesis in pSjD. Senolytic clearance of senescent cells may represent a potential therapeutic strategy for mitigating glandular dysfunction.

**Supplementary Information:**

The online version contains supplementary material available at https://doi.org/10.1186/s12979-026-00590-8.

## Background

Primary Sjögren’s disease (pSjD) is a systemic autoimmune disease primarily affecting exocrine glands, most notably the salivary and lacrimal glands. Clinically, it is characterized by the classic sicca complex, comprising xerostomia and xerophthalmia, which notably draw the attention of dentists and ophthalmologists [1]. Beyond these sicca symptoms, pSjD presents a complex clinical challenge with systemic manifestations ranging from debilitating fatigue to severe extraglandular manifestations like interstitial lung disease [2–4]. Despite its negative impact on quality of life, current therapeutic interventions remain largely palliative, failing to address the underlying disease mechanisms and thus leaving an urgent, unmet clinical demand for precision-based therapeutic strategies.

Epidemiologically, pSjD exhibits a marked sex and age predilection. Two cross-sectional studies reported that female patients accounted for 93.2% and 93.5% of the study populations, with mean diagnostic ages of 53.2 ± 14.2 and 52.2 ± 14.6 years, respectively [5, 6]. The prevalence increases with age, highlighting aging as a crucial risk factor for pSjD [7]. At the molecular level, aging is closely linked to cellular senescence. The persistent accumulation of senescent cells (SnCs) drives age-related diseases, including osteoarthritis [8], osteoporosis [9], Alzheimer’s disease [10], and type 1 diabetes [11]. Given the close association between SnCs and various pathologies, we hypothesized that the accumulation of SnCs might serve as a key driver of glandular dysfunction and persistent autoimmunity in pSjD.

Meanwhile, fibroblasts are now appreciated as a heterogeneous stromal population, with distinct subsets exerting divergent functions in inflammation, tissue remodeling, and immune cell crosstalk. Nevertheless, whether specific senescent fibroblast subpopulations emerge in the salivary glands of patients with pSjD, and how such cells might mediate immune cell infiltration and glandular dysfunction, remains largely undefined. Addressing this knowledge gap is critical for understanding how aging-related mechanisms contribute to pSjD pathogenesis.

By integrating single-cell RNA sequencing (scRNA-seq) with clinical specimen validation, this study systematically mapped the senescence landscape of salivary gland tissues in pSjD. The analysis revealed that the DNAJB1^high^ fibroblast subpopulation constituted the primary senescent cell cluster. In non-obese diabetic (NOD) mice, the administration of senolytic therapy effectively cleared senescent cells, significantly attenuated lymphocyte infiltration, and reversed the decline in salivary secretion.

Further investigation into the molecular mechanisms confirmed that the transcription factor JUND enhanced the transcription of DNAJB1 by directly binding to its promoter, thereby activating the p21-mediated cellular senescence program. These findings linked the senescent cell burden to the development of glandular dysfunction and identified the JUND-DNAJB1-p21 expression axis as a previously unrecognized pathway associated with salivary gland damage in pSjD.

This study not only elucidated the pathological basis for the senescent alterations and accumulation of salivary gland fibroblasts in patients with pSjD, but also demonstrated that the targeted elimination of these senescent cells can partially restore glandular function. Consequently, it provides a novel pharmacological target and transcriptional intervention strategy for the implementation of precision therapy.

## Materials and methods

### Study approval

All animal experiments were conducted at the Center for Animal Experiment/Animal Biosafety Level-III laboratory of Wuhan University. All animal experiments were approved by the Institutional Animal Care and Use Committee (IACUC) of Wuhan University Center for Animal Experiment (No. WP20250015) and followed ARRIVE Guideline 2.0. Human samples were obtained from clinical biopsies. All patients fulfilled the 2016 American College of Rheumatology/European League Against Rheumatism (ACR/EULAR) classification criteria for pSjD [12]. The clinical characteristics of the pSjD patients and healthy controls, including age, sex, and labial gland focus score, are summarized in Supplementary Table 2. The research involving human subjects was conducted in accordance with the Declaration of Helsinki. Written informed consent was obtained from all subjects and ethical approval was granted by Ethics Committee of School & Hospital China Hospital of Stomatology Wuhan University (No. WDKQ2025B33).

### Sex as a biological variable

Our study exclusively examined female mice because the NOD mouse inherently demonstrates a female sex bias in disease penetrance [13]. All human samples were derived from female donors, consistent with the marked female bias in pSjD epidemiology [5].

### Pseudotime analysis

Cellular trajectories and transitional dynamics among clusters were reconstructed using Monocle2 R package (version 2.30.1) with the DDR-Tree algorithm [14]. Branch expression analysis modeling (BEAM) was applied to identify key genes driving lineage-specific fate decisions along the pseudotime.

### RNA velocity

To estimate RNA velocity, we quantified spliced and unspliced transcripts from aligned BAM files using the velocyto python package (version 0.17.17) [15]. Downstream analysis, including velocity vector estimation and low-dimensional embedding, was performed via the scVelo python package (version 0.2.5) [16]. Cell state transitions were modeled using the scVelo transition matrix, with cellular destinations inferred based on maximum correlation values within the matrix.

### PAGA analysis

We employed the PAGA (Partition-based Graph Abstraction) algorithm within the scVelo framework to map the developmental relationships between cell clusters. By applying a statistical model to evaluate cell-cluster interactions, PAGA established connectivity edges between nodes where transcriptomic similarity significantly exceeded stochastic expectations [17].

### SCENIC analysis

To infer gene regulatory networks and identify master regulators at the single-cell level, we performed Single-Cell rEgulatory Network Inference and Clustering (SCENIC) analysis using the pyscenic python package (version 0.12.1) [18]. Co-expression modules identified by GENIE3 were filtered for cis-regulatory motifs using RcisTarget to define regulons. Regulon activity in each cell was then scored using the AUCell algorithm.

### Prediction of transcription factors (TFs)

To identify potential upstream TFs regulating DNAJB1 expression, we utilized the TF-Target Finder tool [19]. The prediction was conducted by integrating data from five independent TF-target interaction databases, including JASPAR, hTF target, ChIP Atlas, GTRD, and ENCODE. We screened for TFs that were consistently predicted across these databases. Candidates identified in the intersection of these datasets were considered high-confidence upstream regulators of DNAJB1 and were selected for further investigation.

### Prediction of JUND binding sites within the DNAJB1 promoter

The promoter sequence of DNAJB1 (Gene ID: 3337), defined as the region (2,000 bp upstream to 100 bp downstream) relative to the transcription start site (TSS), was retrieved from the National Center for Biotechnology Information. The JASPAR database was used to scan for JUND binding motifs with a relative profile score threshold of 80% [20].

### In Silico Molecular Docking

Molecular docking between human JUND (UniProt ID: P25685) and DNAJB1 promoter was performed using AlphaFold 3 via the publicly available server (www.alphafoldserver.com) [21]. Figures depicting the docked complex and interaction schematics were generated using PyMOL Molecular Graphics System v3.0 (Schrödinger LLC).

### Gene perturbation modeling

To predict the regulatory impact of JUND on fibroblast state transitions, we performed in silico perturbation simulations using CellOracle [22]. A tissue-specific gene regulatory network (GRN) was first reconstructed leveraging our single-cell transcriptomic profiles. Based on this contextual GRN, we simulated JUND knockout and overexpression to visualize projected shifts in cell trajectories and identity dynamics. All analyses were executed following the official CellOracle computational framework with default parameters.

### Cell culture

Tissues were obtained from the surgical margins of salivary gland cyst excisions (*n* = 3). The collected specimens were immediately immersed in ice-cold PBS containing 50% triple antibiotics and extensively rinsed. The tissues were then pulverized to a size of approximately 1 mm² and homogenized in culture medium to create a tissue suspension. The fragments were evenly distributed onto the bottom surface of cell culture dishes and cultured in DMEM high-glucose medium (C11995500BT, Gibco) supplemented with 20% FBS (C04002, VivaCell) and 1% triple antibiotics at 37 °C with 5% CO_2_. Fibroblast outgrowth from tissue explants can typically be observed within 5–7 days. Primary fibroblasts were isolated and their purity was verified by immunofluorescence staining. Upon reaching 80–100% confluence, cells were passaged using 0.25% trypsin-EDTA (25200056, Gibco) and maintained in DMEM high-glucose medium containing 10% FBS and 1% triple antibiotics. Fibroblasts at passages 2–5 were used for subsequent experiments.

Jurkat T cells (Cowin Biotech, China) were cultured in RPMI 1640 medium (C11875500BT, Gibco) supplemented with 10% FBS and 1% triple antibiotics under standard conditions (37 °C, 5% CO_2_). Jurkat T cells were activated at an initial density of 1 × 10⁶ cells/mL in complete medium containing 1 µg/mL anti-CD3 (65569-1-MR, Proteintech) and 2 µg/mL anti-CD28 (65099-1-Ig, Proteintech) for 72 h, then maintained in complete medium supplemented with 20 ng/mL interleukin-2 (IL-2; HZ-1015, Proteintech) for subsequent experiments.

### Animal study

The Institute of Cancer Research (ICR) and NOD mice (female, 10-week-old) purchased from the Hubei Provincial Center for Disease Control and Prevention were used as control and pSjD model groups, respectively. The ICR mice served as normal controls, while NOD mice were randomly divided into three experimental groups: untreated group, vehicle treatment group, and dasatinib and quercetin (D&Q) treatment group. Each group consisted of six mice. During the experimental period, all mice were monitored daily for general health status and weighed weekly. Following completion of the experiments, all mice were euthanized and submandibular gland tissues were collected. Half of the samples were processed for Western blot analysis, with the remaining half fixed in 4% paraformaldehyde for subsequent histological examination.

### Immunofluorescent staining

Fibroblasts were seeded in confocal dishes and cultured overnight in complete medium at 37 °C with 5% CO_2_. Cells were fixed with 4% PFA for 15 min, permeabilized with 0.1% Triton X-100 (C-0089, Bioss) for 15 min, and blocked with goat serum (ZLI-9022, ZSGB-Bio) for 1 h at room temperature. Cells were incubated with primary antibody at 4 °C overnight, and then incubated with secondary antibody for 1 h at room temperature, protected from light. Nuclei were stained with DAPI (P0131, Beyotime Biotechnology). Images were acquired using a confocal microscope (SpinSR10, Olympus) and processed with Fiji.

For tissue multiplex immunofluorescence staining, after deparaffinization, the tissue sections were subjected to antigen repair in 1× EDTA antigen retrieval solution (C1033, Solarbio) at 95 °C. After natural cooling to room temperature, the sections were blocked with 3% BSA at room temperature for 30 min. Subsequent steps were the same as above.

Primary antibodies used for immunofluorescence included anti-PDGFRα (84383-2-RR, Proteintech), anti-Vimentin (bs-0756R, Bioss), anti-DNAJB1 (13174-1-AP, Proteintech), anti-p21 (10355-1-AP, Proteintech), anti-CD3 (ab16669, Abcam), and anti-CD4 (ab133616, Abcam). Fluorescent secondary antibodies included 594-conjugated Goat anti-Rabbit IgG (H + L) (AS039, ABclonal), 488-conjugated Goat anti-Rabbit IgG (H + L) (AS073, ABclonal) and triple color multiple fluorescence immunohistochemical staining kit (abs50089, Absin).

### Quantification of immunostaining

For the quantification of p16- and p21-positive cells in human labial gland IHC staining, positive cells were counted in randomly selected regions within the salivary gland lobules for both pSjD and healthy control samples. The number of positive cells was expressed as a percentage of the total number of cells within these random intra-lobular regions. For murine submandibular gland staining, the percentages of p21-, Ki-67-, DNAJB1-, and JUND-positive cells were calculated based on the total number of cells in random fields of view, rather than being restricted to the fibroblast population. All quantification was performed using Fiji software (NIH).

### Cell drug administration

Primary fibroblasts were seeded in 6-well plates and cultured overnight in complete medium at 37 °C with 5% CO_2_. Fibroblasts were treated with varying concentrations of IFN-γ (100–1000 ng/mL, HY-P7025, MedChemExpress) for 72 h to establish an in vitro pSjD model, with untreated cells serving as the control group.

### Staining for senescence-associated galactosidase (SA-β-gal)

Cells were stained for SA-β-gal activity using a commercial kit (C0602, Beyotime Biotechnology) following the instructions. After overnight incubation at 37 °C (12 h, CO_2_-free), images were taken with a light microscope (CKX53, Olympus). The percentage of positive cells was calculated by analyzing six random fields per group using Fiji software (NIH).

### Detection of intracellular reactive oxygen species (ROS)

Intracellular ROS levels were measured using the DCFH-DA fluorescent probe (S0033S, Beyotime Biotechnology). Primary fibroblasts were seeded in 6-well plates and induced into an pSjD model. Cells were incubated with 10 µM DCFH-DA (1 mL/well) at 37 °C for 20 min and were protected from light, followed by three PBS washes. Nuclei were counterstained with Hoechst (C1025, Beyotime Biotechnology) at 37 °C for 20 min. Images were acquired using a confocal microscope (SpinSR10, Olympus) and analyzed with Fiji software (NIH).

### Generation of DNAJB1 knockdown fibroblasts

To generate DNAJB1 knockdown fibroblasts, four siRNA sequences targeting human DNAJB1 were designed and synthesized (GenePharma). The target sense strand sequences are as follows:

- siDNAJB1-1: 5’- GCCAAUGGUACCUCUUUCATT − 3’
- siDNAJB1-2: 5’- GGCUGUACCAAGAAGAUGATT − 3’
- siDNAJB1-3: 5’- CCCCACAAUAUCUUUAAGATT − 3’
- siDNAJB1-4: 5’- CCGUCGUAUUCAAAGAUGUTT − 3’

A non-targeting siRNA (sequence: 5’- UUCUCCGAACGUGUCACGUTT − 3’) served as a negative control (siNC). Transfection was performed according to the manufacturer’s instructions, and transient knockdown cells were obtained after 24 h. Knockdown efficiency was verified by Western blot.

### Co-immunoprecipitation (Co-IP) assay

Co-IP assays were performed using the Pierce™ Immunoprecipitation Kit (26149, ThermoFisher Scientific) following the manufacturer’s protocol. SGFs were lysed with IP Lysis Buffer containing protease inhibitors. 5 µg of anti-DNAJB1, anti-p21 antibodies, and anti-IgG control (30000-0-AP, Proteintech) were covalently coupled to the resin. Total protein lysates (1000 µg) were incubated with the coupled resin for 2 h at room temperature with gentle rotation. Following washing, bound proteins were eluted and analyzed by Western blot to confirm the reciprocal co-precipitation of DNAJB1 and p21.

### In vitro transwell T cell migration assay

To obtain fibroblast-conditioned medium, primary fibroblasts were cultured with complete medium with or without 1000 ng/mL IFNγ at 37 °C for 72 h. Complete medium was then replaced and the culture continued for 24 h. The medium was collected and concentrated (10×) for subsequent experiments. Transwell cell migration assays were performed using 5 μm 24-well plates (14331, Labselect). Fibroblast conditioned medium (600 µL) was loaded into the lower chamber, and then activated Jurkat T (1 × 10^5^) suspended in 100 µL of medium was placed into the upper chamber. After 24 h of incubation at 37 °C with 5% CO_2_, migrated and non-migrated T cells were collected from the lower and upper chambers, respectively, and counted by a cell counter. The migration rate was obtained by dividing the number of migrated cells by the number of migrated plus unmigrated cells.

### Dual-luciferase reporter assay

To evaluate the regulatory effect of JUND on the DNAJB1 promoter, dual-luciferase reporter assays were conducted. All recombinant plasmids, including the JUND overexpression vector and the DNAJB1 promoter-driven luciferase reporters (wild-type and mutant), were constructed by GenePharma. Briefly, 293T cells were seeded in 12-well plates and cultured until they reached 80% confluence. Co-transfection was performed using Lipofectamine 3000 (TL301, Vazyme), incorporating the specified luciferase reporter, a pRL-TK Renilla luciferase plasmid for internal normalization, and either the JUND expression plasmid or an empty control vector. Following a 48-hour incubation, luciferase activities were quantified with a dual-luciferase detection system (RG027, Beyotime Biotechnology) according to the manufacturer’s protocol.

### Senolytic preparation and administration

Dasatinib (S1021, Selleck) and Quercetin (Q4951, Sigma-Aldrich) were dissolved in a vehicle solution consisting of 40% PEG300 (HY-Y0873, MedChemExpress), 5% Tween 80 (HY-Y1891, MedChemExpress), 5% DMSO (A3672, PanReac Applichem), and 50% PBS (C10010500BT, Gibco). Following body weight measurement, the D&Q treatment group received dasatinib (5 mg/kg) and quercetin (50 mg/kg) via oral gavage, while the vehicle control group was administered an equivalent volume of the vehicle solution based on body weight. Each treatment cycle lasted two weeks, with the drug administered once daily for the first three days. This regimen was repeated for a total of four cycles (Fig. 2A).

#### Saliva secretion assessment

Following body weight measurement, mice were anesthetized by intraperitoneal injection of 1.25% tribromoethanol (15 µL/g, MA0478-2, Meilunbio) and subsequently administered pilocarpine (1 mg/kg, 92-13-7, Macklin) via intraperitoneal injection to stimulate saliva secretion. Pre-weighed cotton balls placed in EP tubes were inserted into the oral cavity for saliva collection over a 5-minute period, after which the cotton balls were returned to their original EP tubes for re-weighing. The salivary flow rate was calculated as the amount of increase in weight (mg)/body weight (g).

### Statistical analysis

Fiji software was used to quantify Western blot and IHC staining. Data are presented as mean ± SEM. Unpaired, 2-tailed Student’s t test, One-way ANOVA, Mann-Whitney U test, and Two-way ANOVA were used. Specific statistical analysis is shown in figure legends. Statistically significant differences were considered at * *p* < 0.05, ** *p* < 0.01, *** *p* < 0.001, and **** *p* < 0.0001. All statistical analyses were performed in Prism (version 10.1.1, GraphPad Software) and the ggpubr R package (version 0.6.0).

The detailed protocols for other experiments are described in supplementary material.

## Results

### Fibroblasts as the major senescent cell type in pSjD salivary glands

**Fig. 1:**
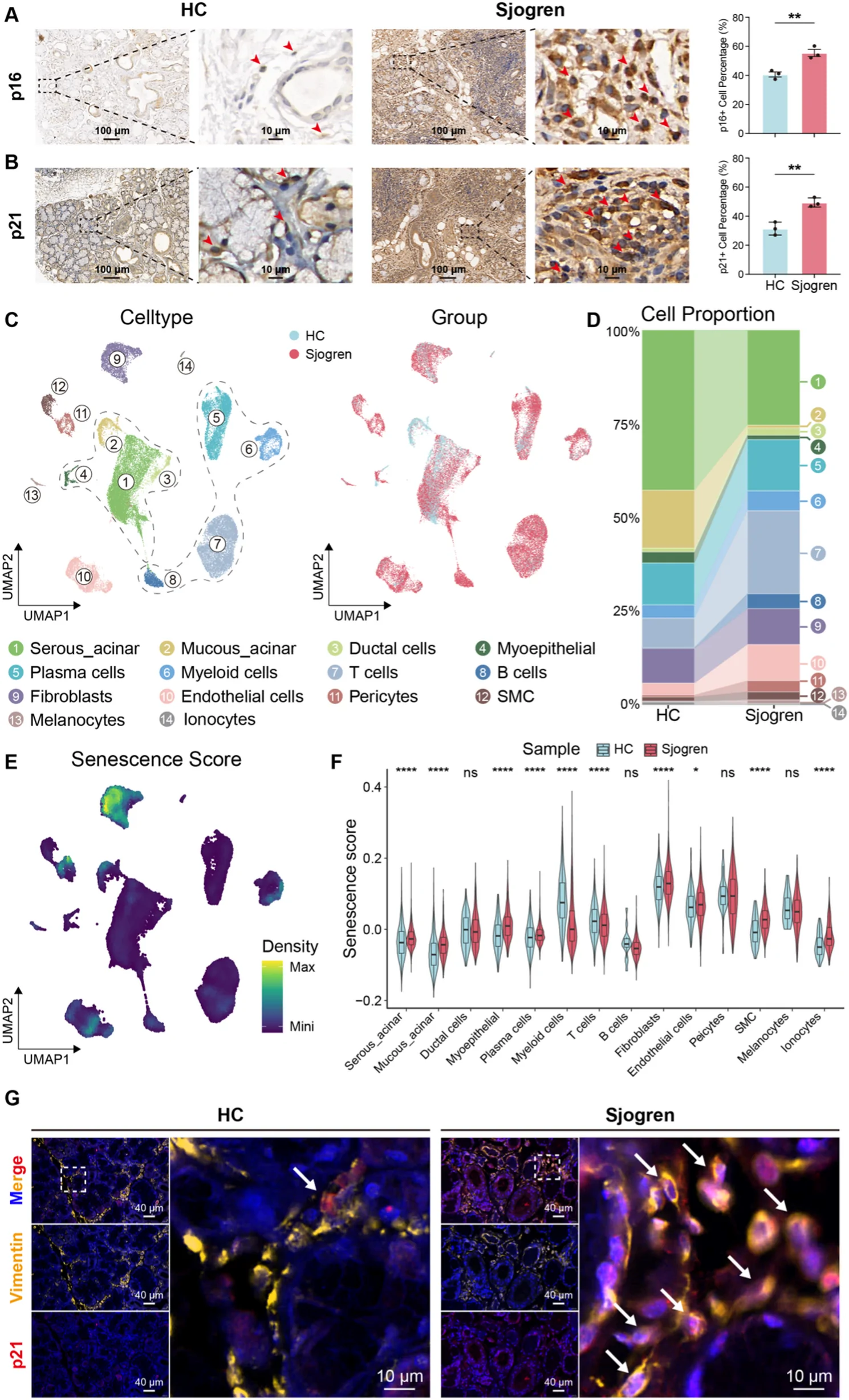
ScRNA-seq analysis revealed fibroblast senescence in pSjD salivary glands. **A-B** Representative IHC staining images illustrate the expression of senescent markers p16 (A) and p21 (B) in labial gland tissues from HC and pSjD patients. Scale bars are 40 μm and 100 μm. The graphs on the right show the quantification of the percentage of p16^+^ and p21^+^ cells in labial glands (*n* = 3). Red arrows indicate representative p16- and p21-positive cells. **C** UMAP plot depicts cells colored by celltype and grouped by disease status. **D** Bar chart shows the proportion of different cell types in HC and pSjD. **E-F** UMAP density plot and violin plot illustrate and statistically compare senescence score between HC (blue) and pSjD (red) groups. **G** Representative mIF images showing p21^+^ fibroblasts in labial glands from HC and pSjD groups. White arrows indicate co-localization of p21 and vimentin. Scale bars are 10 μm and 40 μm, *n* = 3 per group. Unpaired, 2-tailed Student’s t test was used in A, B, and F for statistical analysis. Data are presented as mean ± SEM. * *p* < 0.05, ** *p* < 0.01, *** *p* < 0.001, **** *p* < 0.0001

Given that advancing age is a significant risk factor for the development of pSjD, we first evaluated the accumulation of senescent cells (SnCs) in pSjD salivary glands. Immunohistochemistry (IHC) results revealed significant increases in protein levels of the senescence markers p16 (Fig. 1A) and p21 (Fig. 1B) in the labial gland tissues of pSjD patients compared to healthy controls (HCs), indicating higher SnC accumulation.

To pinpoint the main cell type undergoing senescence, we analyzed scRNA-seq data from labial glands (Fig. 1C, Supplementary Fig. 1). Compared to the HC group, cell proportions in the pSjD group were notably altered, with a significant decrease in epithelial cells (clusters 1–4) and a significant increase in immune cells (clusters 5–8), while the proportion of fibroblasts remained relatively stable (Fig. 1D). Scoring with the SenMayo gene set and the AddModuleScore method revealed fibroblasts to have the highest senescence score among all cell types (Fig. 1E). Furthermore, fibroblasts from the pSjD group showed a significantly higher senescence score compared to those from the HC group (Fig. 1F). This finding was consistently supported by extensive cross-validation using various gene sets and scoring methods (Supplementary Fig. 2). Given the consistency of these results and the high computational efficiency of the AddModuleScore method (which required < 5 s and minimal peak memory; Supplementary Fig. 3), we selected the SenMayo gene set and AddModuleScore for all subsequent analyses.

Finally, multiplex immunofluorescence (mIF) confirmed these results in situ, showing a marked increase in the co-localization of the senescence marker p21 and the fibroblast marker vimentin (p21^+^ vimentin^+^ cells) in pSjD clinical samples (Fig. 1G, indicated by white arrows). Together, these data indicated that salivary gland fibroblasts (SGFs) exhibited senescence in pSjD and might act as key contributors to glandular pathology.

### Senolytic drug-mediated clearance of senescent cells alleviated salivary gland dysfunction in NOD mice

**Fig. 2:**
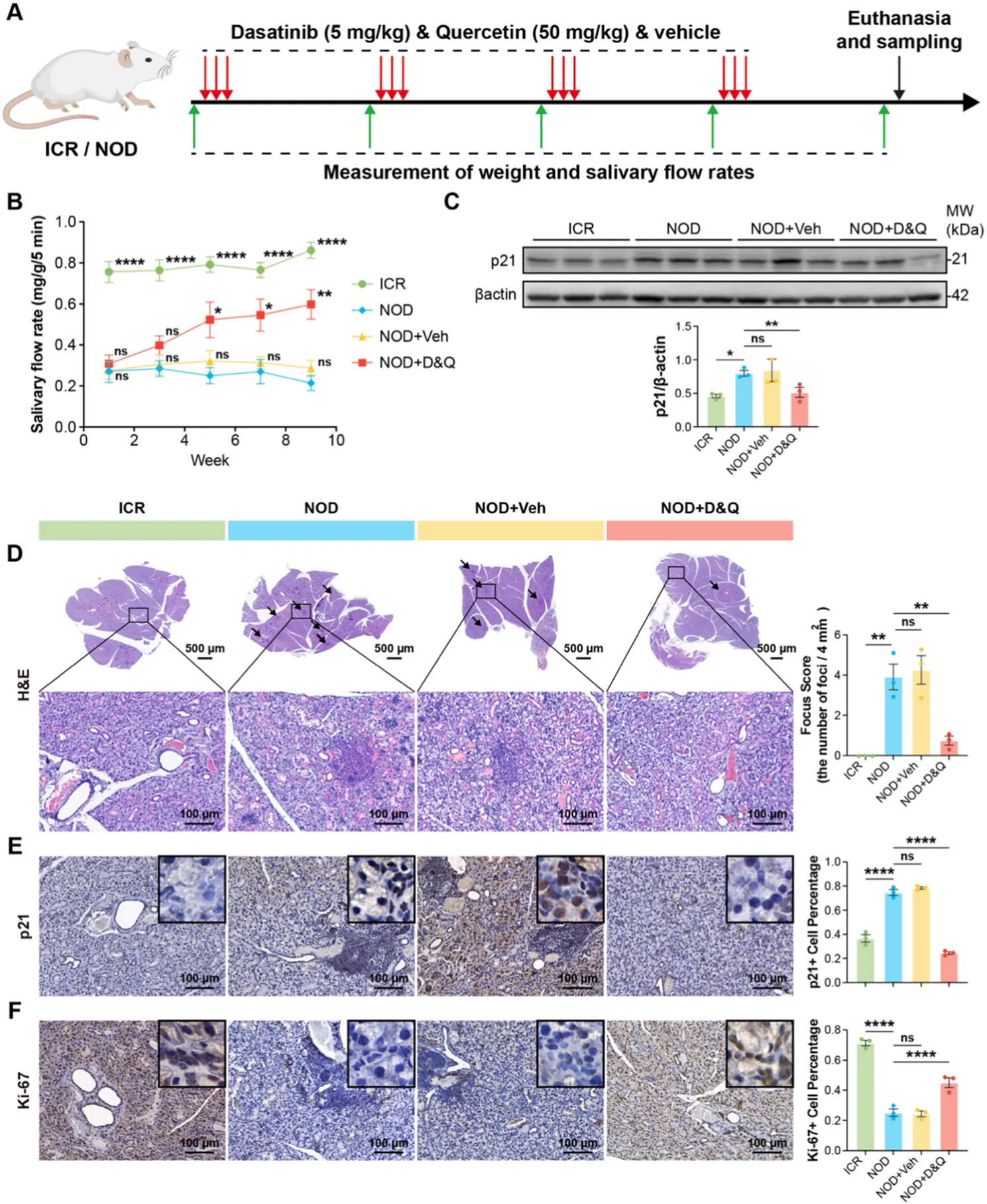
Senolytic drug-mediated clearance of senescent cells alleviated salivary gland dysfunction in NOD mice. **A** Schematic diagram of the experimental design. **B** Line chart showing the measurement of salivary flow rates for four groups (*n* = 6). The other three groups were compared with the NOD group for statistical analysis. **C** Western blot analysis and quantification of p21 protein expression in the salivary glands from the four groups. Band intensities were quantified using Fiji. **D** Representative H&E staining images of submandibular glands from the four groups. The lower row shows a magnified view of the squares in the upper row. The graph on the right shows the quantification of lymphocytic infiltration foci per 4 mm^2^. Scale bars are 100 μm, *n* = 3 per group. **E-F** Representative IHC staining of submandibular glands for p21 (E) and Ki-67 (F). The graphs on the right show the quantification of the percentage of p21^+^ and Ki-67^+^ cells, respectively. Scale bars are 100 μm, *n* = 3 per group. Two-way ANOVA was used in B and One-way ANOVA was used in C-F for statistical analysis. Data are presented as mean ± SEM. * *p* < 0.05, ** *p* < 0.01, *** *p* < 0.001, **** *p* < 0.0001

To determine whether the observed accumulation of SnCs is a bystander effect or a functional contributor to pSjD pathogenesis, we evaluated the therapeutic potential of senescent cell clearance in vivo. We administered the senolytic combination of D&Q or a control vehicle (Veh) via oral gavage to NOD mice. Untreated NOD mice and ICR mice served as positive and negative controls, respectively (Fig. 2A).

Salivary flow rates were measured every two weeks throughout the study. Compared to the NOD and NOD + Veh groups, the NOD + D&Q group showed a significant recovery in salivary flow rates after two treatment courses, indicating a significant alleviation of hyposalivation (Fig. 2B). Western blot analysis confirmed a global reduction in p21 protein levels, and further IHC staining localized this decrease within the salivary gland interstitium of NOD mice (Fig. 2C, E). Focus score (FS) in salivary glands is a key pathological indicator for pSjD with a sensitivity and specificity greater than 80% [23]. We assessed FS through H&E staining. Compared to the NOD and NOD + Veh groups, the NOD + D&Q group showed a significant decrease in FS in the salivary glands (Fig. 2D). Additionally, the protein level of the cell proliferation marker Ki-67 was higher in the NOD + D&Q and ICR groups (Fig. 2F). Multiplex immunofluorescence co-staining with the fibroblast-specific marker PDGFRα confirmed that p21 and Ki-67 were expressed in fibroblasts (Supplementary Fig. 4). These results suggested that clearing SnCs effectively mitigated salivary gland dysfunction, providing a rationale for investigating the specific senescent subpopulations involved.

### DNAJB1^high^ fibroblasts represented the core senescent subpopulation in SGFs

**Fig. 3:**
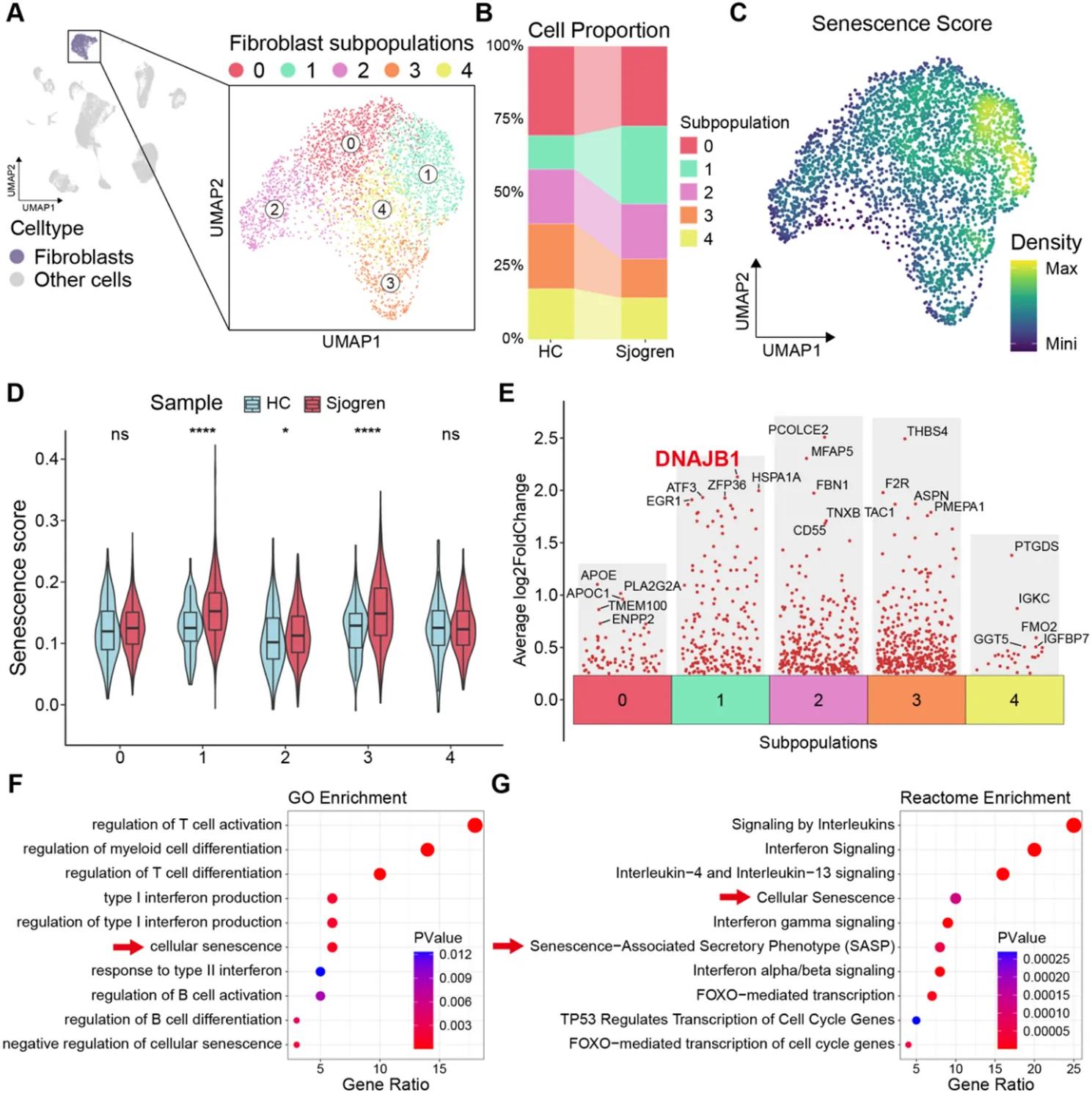
Fibroblast subpopulation 1 represented the core subpopulation of senescent salivary gland fibroblasts. **A** UMAP plot showing the reclustering of fibroblasts (*n* = 3719 cells) into five distinct subpopulations (0–4), colored by subpopulation. **B** Bar chart showing the proportion of each fibroblast subpopulation in HC and pSjD groups. **C-D** UMAP density plot and violin plots show and statistically compare the senescence score across fibroblast subpopulations between the HC (blue) and pSjD (red) groups. **E** Dot plots showing the DEGs of each fibroblast subpopulation and labelling the top five DEGs (based on average log2FoldChange). **F-G** GO enrichment and Reactome pathway enrichment analysis of the DEGs from subpopulation 1. The size of the dot represents the gene count, and the color intensity represents the p-value. Unpaired, 2-tailed Student’s t test was used in D for statistical analysis. Data are presented as mean ± SEM. * *p* < 0.05, ** *p* < 0.01, *** *p* < 0.001, **** *p* < 0.0001

Given that senolytic treatment effectively alleviated salivary gland dysfunction in NOD mice, we sought to characterize fibroblast heterogeneity and identify the subpopulation most closely associated with these effects. Re-clustering of the scRNA-seq data identified five distinct SGF subpopulations (Fig. 3A), among which subpopulation 1 was notably characterized by its significant expansion in pSjD (Fig. 3B, Supplementary Fig. 5). Subsequent senescence scoring and violin plot analysis revealed that subpopulation 1 possessed the highest senescence level, which was markedly further elevated under pSjD conditions (Fig. 3C, D). We then identified the differentially expressed genes (DEGs) for each subpopulation, highlighting the top five DEGs with the highest average log2FoldChange (Fig. 3E). Gene Ontology (GO) and Reactome pathway enrichment analyses of subpopulation 1’s DEGs confirmed that subpopulation 1 was characterized by the upregulation of cellular senescence, senescence-associated secretory phenotype (SASP), and IFN-γ signaling pathways (Fig. 3F, G). These data indicated that subpopulation 1 was the predominant senescent subpopulation, which might play a crucial role in the pathological progression of pSjD.

**Fig. 4:**
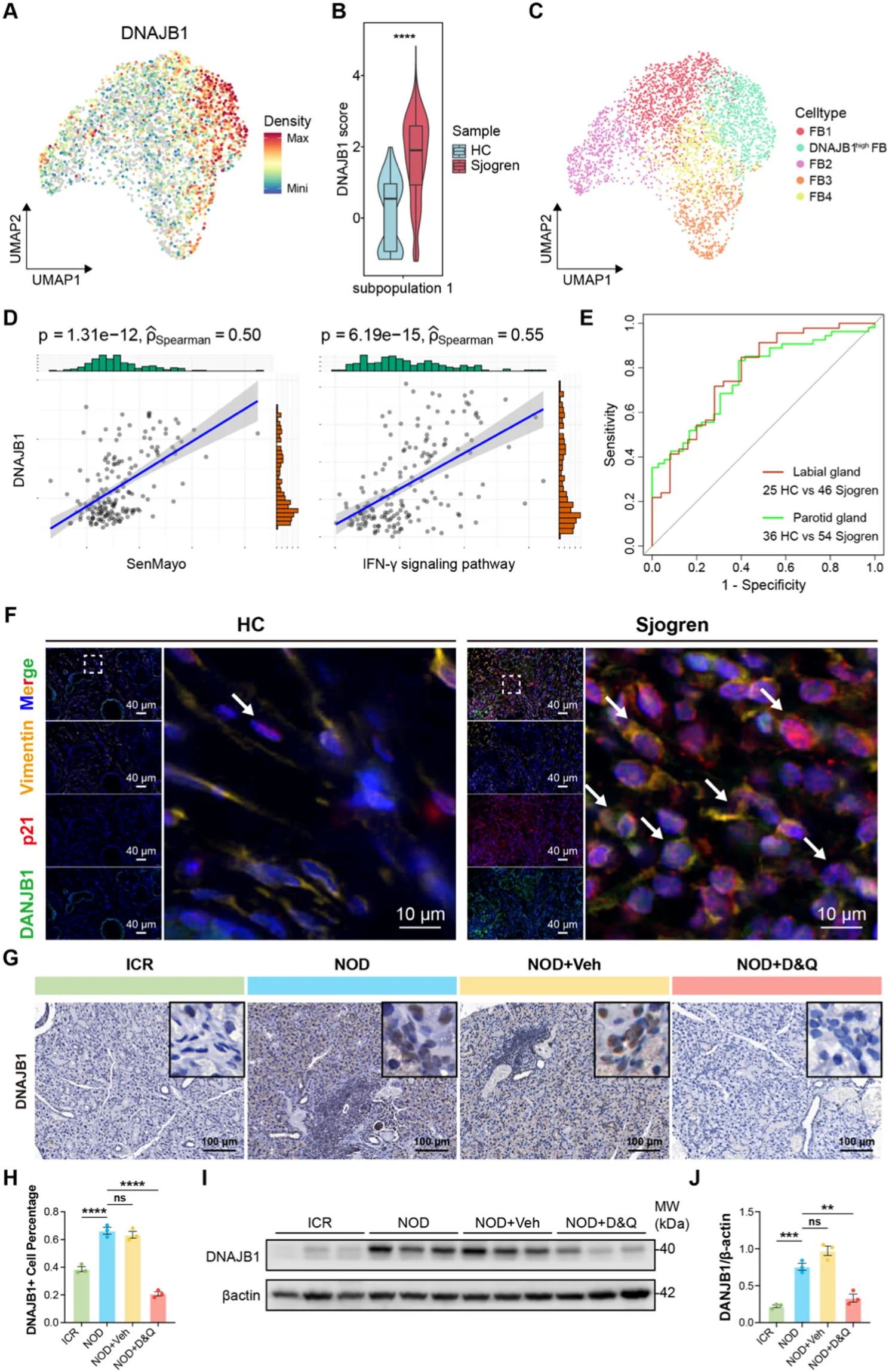
DNAJB1 marked the core senescent fibroblast subpopulation in pSjD. **A-B** UMAP density plot and violin plots show and statistically compare the DNAJB1 score across fibroblast subpopulations between the HC (blue) and pSjD (red) groups. **C** UMAP plot showing the re-annotation of fibroblast subpopulations. **D** Scatter plots showing the correlation between the DNAJB1 score, the senescence score, and the IFN-γ signaling pathway score. The Spearman’s correlation coefficient (ρ) and p-value are indicated. **E** ROC curves showing the diagnostic performance of a panel of four genes (CDKN1A, CDKN2A, DNAJB1, and VIM) for distinguishing pSjD from HC in both labial and parotid glands. **F** Representative mIF staining images of p21^+^ DNAJB1^+^ fibroblasts in the labial glands of HC and pSjD groups. The white arrows indicate the co-localization of p21, DNAJB1, and vimentin. Scale bars are 10 μm and 40 μm, *n* = 3 per group. **G** Representative IHC staining of submandibular glands for DNAJB1. Scale bars are 100 μm, *n* = 3 per group. **H** The graph shows the quantification of the percentage of DNAJB1^+^ cells. **I-J** Western blot analysis and quantification of DNAJB1 protein expression in the salivary glands from the four groups. Band intensities were quantified using Fiji. Unpaired, 2-tailed Student’s t test was used in B for statistical analysis. Data are presented as mean ± SEM. * *p* < 0.05, ** *p* < 0.01, *** *p* < 0.001, **** *p* < 0.0001

To identify a molecular marker highly enriched in subpopulation 1, we focused on the top-ranked molecule DNAJB1. The spatial distribution of DNAJB1 score highly paralleled that of senescence score, with subpopulation 1 exhibiting a significant enrichment of DNAJB1 in pSjD (Fig. 4A, B). Cells in subpopulation 1 were then categorized as DNAJB1^high^ fibroblasts (Fig. 4C). Considering the established role of immune-derived IFN-γ in pSjD pathogenesis [24, 25], we evaluated the correlations between DNAJB1 expression, the SenMayo gene set, and IFN-γ signaling. The results showed strong positive correlations among them, with Spearman’s ρ values of 0.50 and 0.55, respectively (Fig. 4D). Cell-cell communication analysis revealed that DNAJB1^high^ fibroblasts were the primary responders to IFN-γ signaling (Supplementary Fig. 6). Furthermore, receiver operating characteristic (ROC) curves demonstrated that DNAJB1^high^ senescent SGFs had high discriminatory power for pSjD in both labial and parotid glands (Fig. 4E, AUC = 0.7757 and AUC = 0.7608). This indicated that DNAJB1 was highly enriched in, and closely associated with, the senescent SGF subpopulation.

Multiplex immunofluorescence (mIF) results further validated our findings, showing marked increases in both DNAJB1 and p21 protein levels in SGFs from pSjD tissues. There was also a high degree of co-localization among vimentin, DNAJB1, and p21 (Fig. 4F, indicated by white arrows). To more precisely characterize the fibroblast population, we performed additional mIF staining using the fibroblast-specific marker PDGFRα, which consistently demonstrated its co-localization with DNAJB1 and p21 (Supplementary Fig. 7, indicated by white arrows). Consistently, IHC and Western blot analyses of animal experiments also revealed a decrease in DNAJB1 expression after the clearance of senescent cells (Fig. 4G-J). Collectively, these findings suggested that the DNAJB1^high^ fibroblast subpopulation might play a key regulatory role in the progression of pSjD.

### Pseudotime and RNA velocity revealed fibroblast trajectory toward a DNAJB1^high^ phenotype

**Fig. 5:**
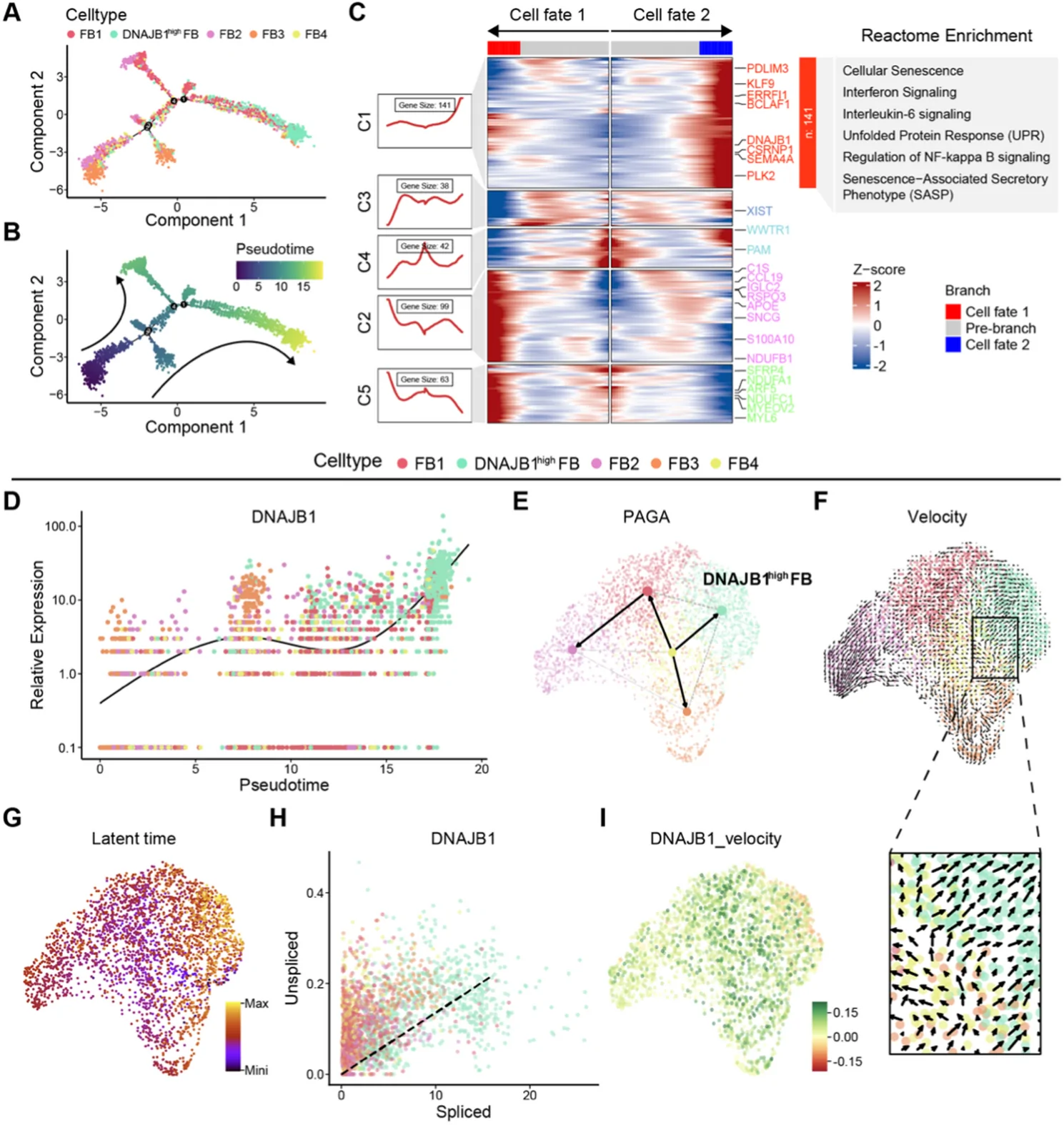
Differentiation dynamics of DNAJB1^high^ fibroblasts. **A** Monocle trajectory of fibroblast subpopulations colored by cell type. **B** Monocle trajectory of fibroblast subpopulations colored by pseudotime. **C** Heatmap of gene dynamics toward two cell fates. Left: Expression trends for modules C1-C5. Right: Representative genes and reactome enrichment for module C1. **D** Scatter plot showing the relative expression of DNAJB1 across pseudotime. Points are colored by cell subpopulation. **E** PAGA graph showing subpopulation connectivity. **F** Projection of RNA velocity streamlines onto UMAP plot. Arrows indicate the predicted future state of cells. The bottom inset shows a magnified view of the velocity vectors. **G** Latent time projected onto the UMAP plot. **H-I** RNA velocity kinetics for the DNAJB1 gene. **H** Phase portrait showing the relationship between unspliced and spliced mRNA abundance. **I** DNAJB1-specific velocity values projected onto the UMAP plot

To investigate the developmental relationship among fibroblast subpopulations, we performed pseudotime trajectory analysis. Pseudotime analysis predicted that DNAJB1^high^ fibroblasts predominantly resided at the terminal end of the trajectory, suggesting a terminally differentiated state (Fig. 5A, B). To further elucidate mechanisms driving divergent cell fates, we identified five gene modules (C1-C5) with distinct dynamics (Fig. 5C). Specifically, genes in module C1, including DNAJB1, exhibited a significant upregulation during the transition toward cell fate 2. Reactome pathway enrichment analysis for C1 genes (*n* = 141) yielded results highly consistent with those of the DEGs in DNAJB1^high^ fibroblasts. These findings suggested that differentiation toward cell fate 2 is linked to cellular stress and the acquisition of a senescence-like state. Furthermore, the relative expression of DNAJB1 showed a progressive increase along the pseudotime, peaking in the DNAJB1^high^ fibroblasts (Fig. 5D).

To validate this developmental trajectory from multiple dimensions, we utilized PAGA to assess the connectivity between subpopulations. The results showed strong topological associations between DNAJB1^high^ fibroblasts and FB4 (Fig. 5E). Subsequently, RNA velocity analysis was employed to predict the directional trends of cell state transitions. The velocity streamlines and the velocity-derived pseudotime both predicted a directional transition toward the DNAJB1^high^ fibroblast state (Fig. 5F, G). Finally, kinetic analysis of DNAJB1 revealed an active transcriptional state in the phase portrait and positive velocity values (Fig. 5H, I), suggesting that DNAJB1 was actively transcribed during this process. Taken together, these computational analyses suggested that DNAJB1^high^ fibroblasts represented a putative terminally differentiated senescent fate in pSjD.

### DNAJB1 mediated IFN-γ-induced senescence in SGFs

**Fig. 6:**
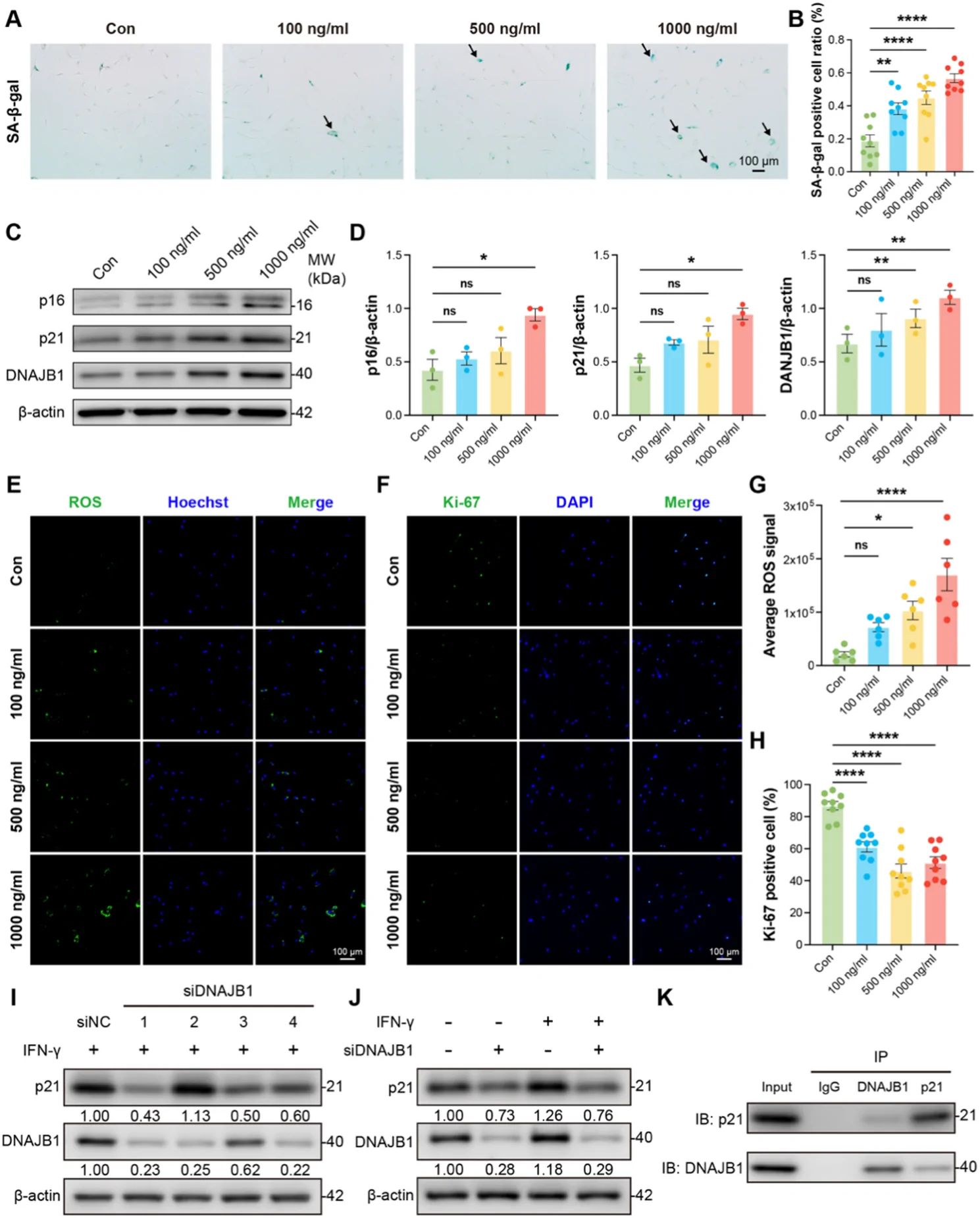
IFN-γ induced SGF senescence via the DNAJB1-p21 axis. **A-B** Representative images and quantification in SGFs treated with different concentrations of IFN-γ. Scale bars are 100 μm. **C-D** Western blot analysis and quantification showing the expression of p16, p21, and DNAJB1 in SGFs treated with different concentrations of IFN-γ. **E** and **G** Representative images and quantification of ROS staining in SGFs treated with different concentrations of IFN-γ. Scale bar is 100 μm. **F** and **H** Representative images and quantification of Ki-67 immunofluorescence staining in SGFs treated with different concentrations of IFN-γ. Scale bar is 100 μm. **I** Western blot analysis showing the knockdown efficiency of four different siRNAs targeting DNAJB1. **J** Western blot analysis showing the protein levels of p21 and DNAJB1 in SGFs with different treatments. **K** Co-IP and Western blot analysis confirming the interaction between DNAJB1 and p21. One-way ANOVA was used in B, D, G, and H. Data are presented as mean ± SEM. * *p* < 0.05, ** *p* < 0.01, *** *p* < 0.001, **** *p* < 0.0001

Building on the computational prediction that DNAJB1^high^ fibroblasts reside at a terminal senescent state, we next investigated the functional involvement of IFN-γ and DNAJB1 in this phenotype in vitro. We extracted primary SGFs (Supplementary Fig. 8) and treated them with different concentrations of IFN-γ (100, 500, and 1000 ng/mL) [26]. The activation of β-galactosidase is an early marker of cellular senescence, typically absent in proliferating or quiescent cells [27]. Senescence-Associated β-Galactosidase (SA-β-gal) staining showed that the proportion of SA-β-gal positive cells increased with increasing IFN-γ concentration (Fig. 6A, B). Furthermore, senescent cells often become enlarged and irregularly shaped due to cell cycle arrest and continuous metabolic activity [28]. Microscopic examination revealed that IFN-γ-stimulated SGFs exhibited these characteristic morphological features of senescent cells (Fig. 6A, indicated by black arrows). Western blot analysis further revealed that the protein levels of p16 and p21 increased in an IFN-γ dose-dependent manner (Fig. 6C, D). Crucially, DNAJB1 protein expression showed a similar dose-dependent upregulation pattern to p16 and p21 (Fig. 6C, D). Since cellular senescence is associated with the accumulation of reactive oxygen species (ROS), we also measured ROS levels. Our results indicated that IFN-γ increased ROS levels in SGFs in a dose-dependent manner (Fig. 6E, G). We then assessed the proliferative capacity of SGFs after IFN-γ induction. Ki-67 immunofluorescence staining showed that IFN-γ treatment reduced SGF proliferation (Fig. 6F, H). These findings indicated that IFN-γ treatment induced SGF senescence and concurrently increased DNAJB1 protein levels.

To investigate the role of DNAJB1, we performed siRNA-mediated knockdown using four siDNAJB1 sequences. Western blot confirmed that DNAJB1 depletion significantly reduced IFN-γ-induced p21 protein levels (Fig. 6I). The fourth siDNAJB1 sequence was found to be the most effective (Fig. 6I), and was selected for subsequent experiments. SGFs with DNAJB1 knockdown significantly attenuated their response to subsequent IFN-γ stimulation (Fig. 6J). To understand the relationship between DNAJB1 and p21, we performed co-immunoprecipitation (Co-IP) assays. The results showed that there was an interaction between DNAJB1 and p21 (Fig. 6K). These experiments indicated that DNAJB1 acted as a critical mediator of IFN-γ-induced senescence.

### IFN-γ treatment enhanced SGFs’ ability to recruit CD4^+^ T cells

**Fig. 7:**
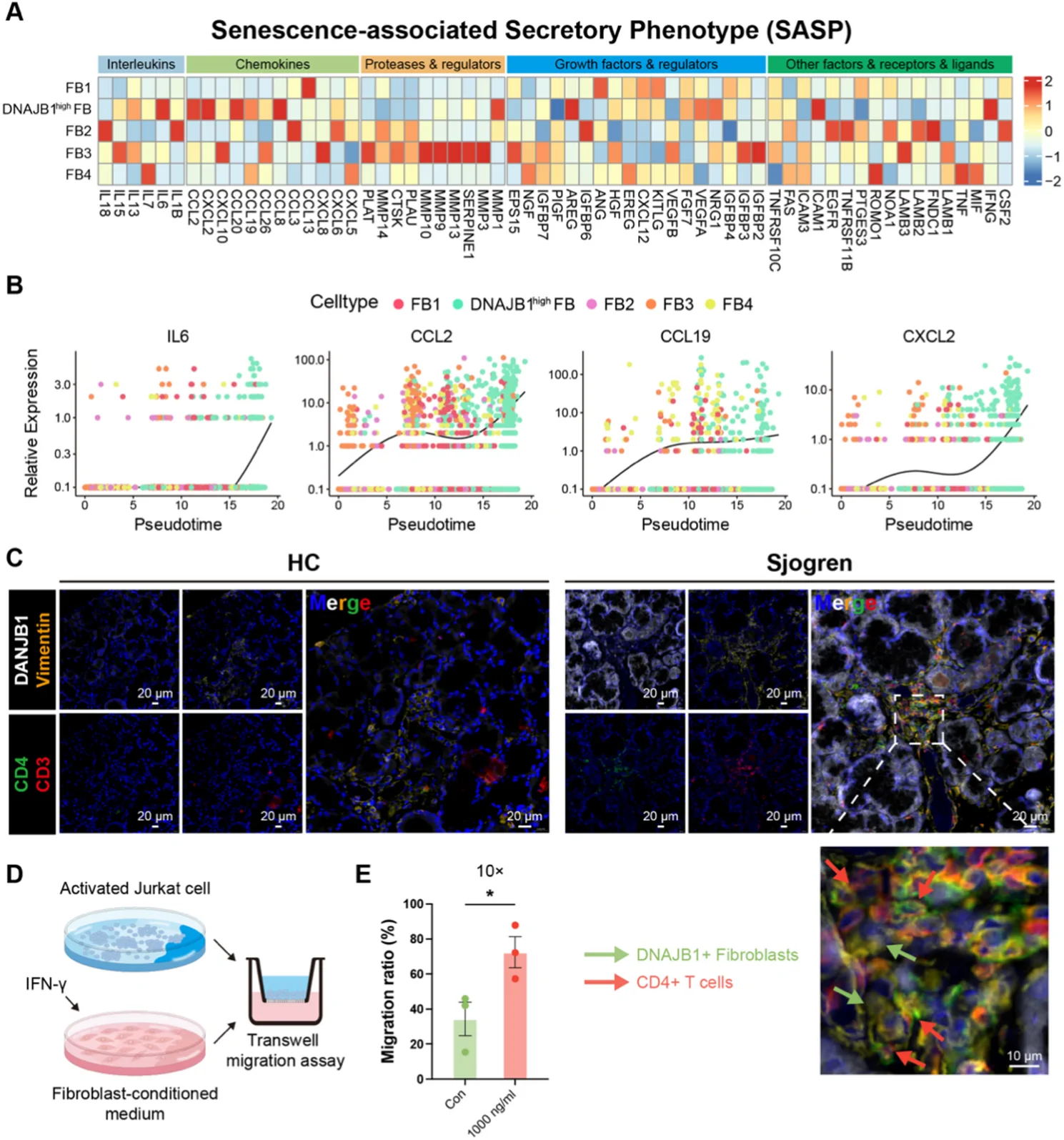
The pro-inflammatory properties of senescent fibroblasts and their role in CD4^+^ T cell recruitment. **A** Heatmap showing the expression of SASP genes in five fibroblast subpopulations. Gene categories include interleukins, chemokines, proteases & regulators, growth factors & regulators, and other factors & receptors & ligands. The color scale indicates the average expression. **B** Scatter plots showing the relative expression of IL6, CCL2, CCL19, and CXCL2 across pseudotime. Points are colored by cell subpopulation. **C** Representative mIF staining images showing DNAJB1^+^ fibroblasts and CD4^+^ T cells in labial glands. A high-power field (bottom) showing close colocalization between DNAJB1^+^ fibroblasts (green arrows) and CD4^+^ T cells (red arrows). Scale bars are 10 μm and 20 μm. **D** Schematic illustration of the Transwell migration assay. **E** Bar chart showing the migration ratio of activated Jurkat T cells towards 10× medium from SGFs. Unpaired, 2-tailed Student’s t test was used in E for statistical analysis. Data are presented as mean ± SEM. * *p* < 0.05, ** *p* < 0.01, *** *p* < 0.001, **** *p* < 0.0001

The reduction of lymphocytic infiltration observed in D&Q-treated mice led us to hypothesize that DNAJB1^high^ fibroblasts modulated the immune microenvironment. The heatmap showed that DNAJB1^high^ fibroblasts expressed high levels of SASP-related genes, particularly key chemokines such as IL6, CCL2, CCL19, and CXCL2 (Fig. 7A). Notably, the expression of these markers increased progressively along the pseudotime trajectory, peaking in the DNAJB1^high^ fibroblasts (Fig. 7B).

Given these molecular findings, we next investigated the immune landscape of pSjD salivary glands. The scRNA-seq data identified T cells as the most abundant immune population, accounting for approximately 49.03% of total immune cells (Fig. 1D). Subpopulation analysis identified CD4^+^ and CD8^+^ T cells as the two major subsets (Supplementary Fig. 9A, B), with a higher proportion of CD4^+^ T cells in pSjD patients (Supplementary Fig. 9C). To verify the functional link between DNAJB1^high^ fibroblasts and CD4^+^ T cell infiltration, we first performed mIF staining on clinical samples. The results revealed that CD4^+^ T cells were extensively enriched in pSjD salivary glands and exhibited close spatial proximity to DNAJB1^high^ fibroblasts (Fig. 7C, Supplementary Fig. 10). We further employed an in vitro T cell transwell migration assay (Fig. 7D) to examine whether DNAJB1^high^ fibroblasts actively recruit CD4^+^ T cells. The results demonstrated that conditioned medium from IFN-γ-treated SGFs significantly enhanced the chemotactic migration of CD4^+^ T cells (Fig. 7E). These findings indicated that DNAJB1^high^ fibroblasts secreted high levels of chemokines and recruited CD4^+^ T cells for tissue infiltration.

### D&Q inhibited DNAJB1-mediated senescence by suppressing JUND-dependent transcription

**Fig. 8:**
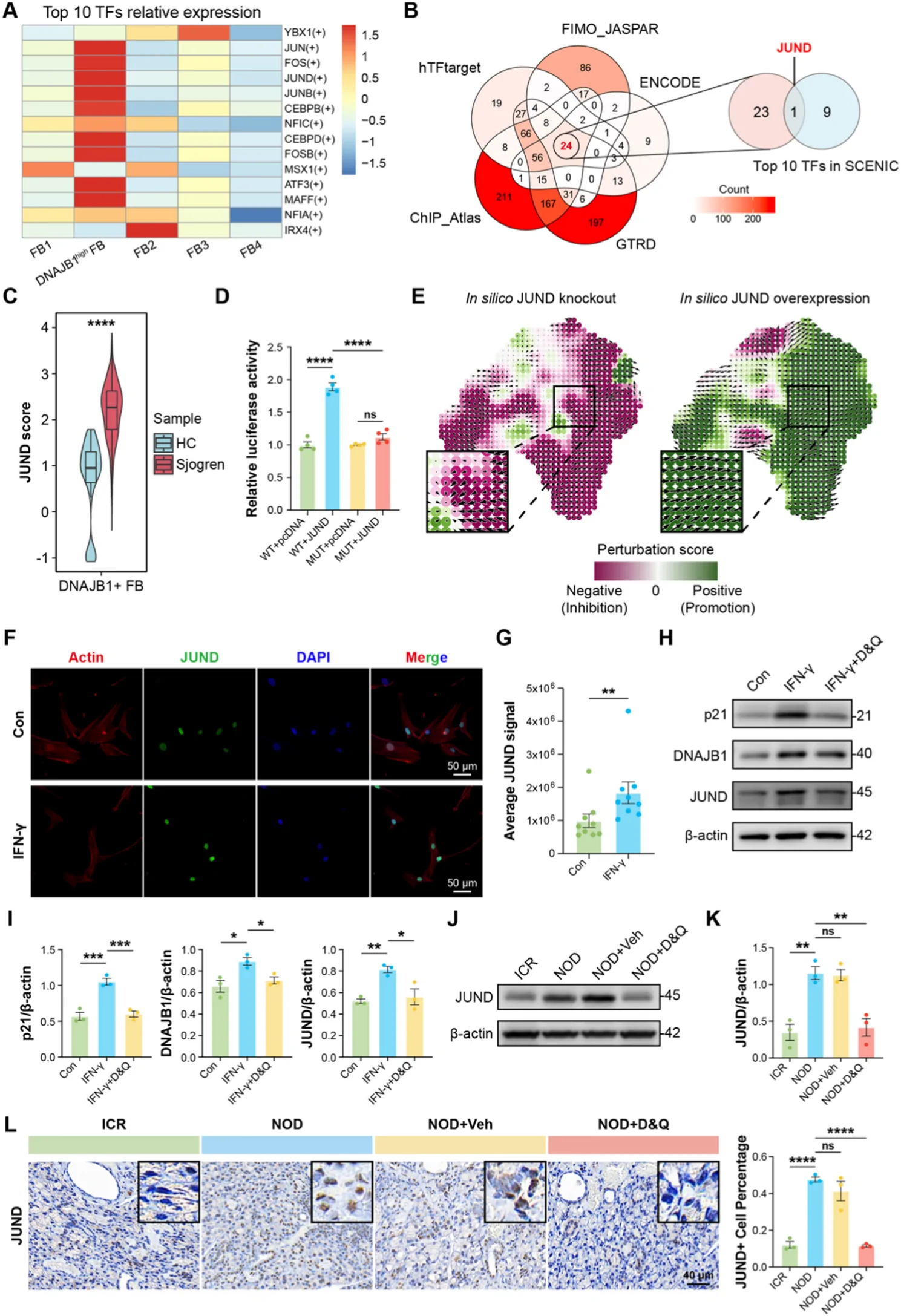
Senolytic drug D&Q inhibited DNAJB1-mediated senescence by suppressing JUND-dependent transcription. **A** SCENIC analysis heatmap showing TFs enrichment in fibroblast subpopulations. **B** Venn diagram intersects SCENIC results with five databases (JASPAR, hTF target, ChIP Atlas, GTRD, and ENCODE) to pinpoint JUND. **C** Violin plot comparing JUND score in DNAJB1^high^ fibroblasts of HC and pSjD groups. **D** Dual-luciferase reporter assays showing that the JBS mutation abrogated capacity of JUND to act on the DNAJB1 promoter region. **E** Predicted shifts in fibroblast trajectories following in silico knockout and overexpression of JUND, modeled by CellOracle. **F-G** Representative images and quantification of JUND immunofluorescence staining in SGFs. Scale bar is 50 μm. **H-I** Western blot analysis and quantification of the expression levels of p21, DNAJB1, and JUND in SGFs subjected to different treatments. **J-K** Western blot analysis and quantification of JUND protein expression in the salivary glands from the four groups. **L** Representative IHC staining of submandibular glands for JUND. The graphs on the right shows the quantification of the percentage of JUND^+^ cells. Unpaired, 2-tailed Student’s t test was used in C and G for statistical analysis. One-way ANOVA was used in D, I, K, and L. Data are presented as mean ± SEM. * *p* < 0.05, ** *p* < 0.01, *** *p* < 0.001, **** *p* < 0.0001

Finally, we investigated the upstream transcriptional mechanisms driving the DNAJB1^high^ phenotype and how D&Q interferes with this process. We utilized SCENIC analysis and inferred high activity of the AP-1 family (including JUN, FOS, JUND, JUNB, and FOSB) in DNAJB1^high^ fibroblasts (Fig. 8A). By intersecting regulatory predictions from five databases (JASPAR, hTF target, ChIP Atlas, GTRD, and ENCODE) with the SCENIC data, we predicted JUND as a core candidate directly regulating DNAJB1 transcription (Fig. 8B). Notably, the JUND score was significantly upregulated in the DNAJB1^high^ subpopulation of the pSjD group (Fig. 8C) and exhibited strong positive correlations with the IFN-γ signaling pathway score (Spearman’s ρ = 0.55), the DNAJB1 score (Spearman’s ρ = 0.84) and the senescence score (Spearman’s ρ = 0.45) (Supplementary Fig. 11A). Mechanistically, motif analysis using the JASPAR database identified only a single putative JUND-binding site (JBS) with a score exceeding 10 within the DNAJB1 promoter (Supplementary Fig. 11B), and AlphaFold3 modeling predicted a high-confidence interaction between JUND and this site (Supplementary Fig. 11C). Furthermore, dual-luciferase reporter assays combined with site-directed mutagenesis of this JBS revealed that the mutation completely abolished the ability of JUND to upregulate DNAJB1 transcription (Fig. 8D). To investigate how JUND orchestrates fibroblast differentiation, we employed CellOracle to predict cell-state dynamics under JUND perturbation. The simulation indicated that JUND deficiency impeded the development of DNAJB1^high^ fibroblasts, whereas JUND overexpression promoted this lineage transition (Fig. 8E). These computational and experimental findings supported an important role of JUND in regulating the DNAJB1^high^ fibroblast state during pSjD pathogenesis.

In vitro immunofluorescence assays revealed a marked increase in nuclear JUND expression in SGFs following IFN-γ stimulation (Fig. 8F, G). Using an optimized senolytic regimen (10 nM dasatinib and 20 µM quercetin), which yielded maximal p21 reduction (Supplementary Fig. 12), we found that D&Q treatment effectively reversed IFN-γ-induced JUND upregulation and concurrently suppressed downstream DNAJB1 and p21 levels (Fig. 8H, I). Consistently, D&Q administration in NOD mice significantly reduced glandular JUND expression, as confirmed by both Western blot and IHC analyses (Fig. 8J-L). Multiplex immunofluorescence further localized expression of DNAJB1 and JUND within glandular regions to fibroblasts (Supplementary Fig. 13). These results indicated that D&Q attenuated salivary gland pathology in association with suppression of JUND-mediated transcriptional activation of the DNAJB1-p21 senescence pathway.

## Discussion

Currently, therapeutic options for primary Sjögren’s disease (pSjD) are limited to symptomatic management and systemic immunosuppressive therapies, with no targeted therapies [29–32]. Moreover, these therapies pose significant challenges due to associated side effects; for instance, long-term use of immunosuppressive drugs can cause hepatotoxicity, nephrotoxicity, and reduced anti-infective capacity [30]. This limitation underscores the urgent need for novel therapeutic strategies. Anti-senescence strategies have emerged as novel approaches for treating age-related diseases, demonstrating efficacy across various conditions [10, 11]. Consequently, there is a strong rationale for exploring the role of senescence in pSjD and investigating anti-senescence as a potential therapeutic method.

Earlier studies have characterized the functional heterogeneity of SGFs, identifying pro-inflammatory subpopulations (e.g., CXCL10^+^ CCL19^+^ and IL32^+^ fibroblasts) that drive immune cell crosstalk and tertiary lymphoid structure (TLS) formation [33–36]. Concurrently, cellular senescence is increasingly recognized as a pathogenic factor in various autoimmune diseases, where senescent cells secrete a senescence-associated secretory phenotype (SASP) to propagate local inflammation. However, the exact intersection between fibroblast heterogeneity and cellular senescence in pSjD remains incompletely understood. Specifically, the molecular pathways governing how specific SGF subpopulations acquire a pro-inflammatory senescent state to orchestrate immune infiltration represent an existing knowledge gap. To address this, our study provides evidence that SGF senescence acts as an active contributor to, rather than a mere bystander effect of, the pathological progression of pSjD. Considering senescent SGFs may contribute to the pathogenesis of pSjD, we hypothesized that targeting senescent SGFs could alleviate glandular damage. We validated this therapeutic potential using the senolytic combination D&Q, which is widely studied for various age-related diseases [37, 38]. Our in vivo data demonstrated that oral D&Q administration significantly alleviated glandular dysfunction in pSjD model mice and reduced the focus score in the salivary glands. These findings provide evidence for senolytic therapy as a novel treatment strategy for pSjD.

To further define the identity of these pathogenic cells, we identified DNAJB1 as highly enriched in, and closely associated with, the core senescent SGF subpopulation. DNAJB1 is a member of the heat shock protein 40 (Hsp40) family and acts as a co-chaperone for Hsp70, playing a critical role in regulating protein folding and targeted protein degradation via the ubiquitin-proteasome system [39, 40]. The expression of intracellular Hsp family members is typically triggered by environmental stress through a mechanism known as the heat shock response (HSR) [41]. Notably, hyperactivation of the HSR, which is mediated by the mammalian target of rapamycin (mTOR), has been implicated in various pathological processes, including cellular senescence [42]. The favorable diagnostic performance of the DNAJB1^high^ senescent SGFs in ROC analyses further underscored the potential of DNAJB1 as a clinical biomarker for pSjD-associated glandular damage. Our study also revealed a significant positive correlation between DNAJB1 expression in SGFs and p21-mediated cellular senescence, as supported by siRNA-mediated knockdown and Co-IP assays. It should be noted, however, that while the Co-IP data demonstrate a physical interaction between DNAJB1 and p21, they do not establish direct functional regulation; the precise molecular mechanism by which DNAJB1 sustains p21 expression remains incompletely defined and requires further investigation. Therefore, we postulate that DNAJB1 closely links the stress response to SGF senescence in pSjD.

While previous studies have established that T helper 1 cell-derived IFN-γ contributes to pSjD pathogenesis, the cellular targets of IFN-γ within the glandular microenvironment have been less explored. Mechanistically, our results demonstrated that IFN-γ treatment induced SGF senescence in a dose-dependent manner. To elucidate the upstream transcriptional regulation of this process, we identified the transcription factor JUND as a key regulator. Utilizing the JASPAR database to predict potential binding motifs and validating these interactions via dual-luciferase reporter assays, we confirmed that JUND promoted DNAJB1 transcription by directly binding to its promoter. Notably, treatment of NOD mice with D&Q significantly reduced glandular protein levels of JUND, DNAJB1, and p21, further validating the in vivo relevance of our proposed mechanistic axis. Consequently, our findings identified the JUND-DNAJB1-p21 axis as a key contributor to SGF senescence, delineating a previously unrecognized mechanism by which the inflammatory glandular microenvironment may shape fibroblast fate in pSjD.

We also demonstrated that DNAJB1^high^ SGFs acquired an enhanced ability to recruit CD4^+^ T cells. Given that CD4^+^ T cells have been shown to be the main immune cells infiltrating the salivary glands in pSjD and play an important role in pSjD salivary gland pathology [43–45], our results suggested a vicious cycle. In this context, inflammation drives SGF senescence, and the senescent SGFs, in turn, sustain chronic inflammation and pathology through T cell recruitment and the secretion of SASP factors, particularly chemokines like CCL2. Consistent with this, existing research indicates a clear correlation between CCL2^high^ fibroblasts and both immune infiltration and disease activity in pSjD [46]. This underscores the importance of the DNAJB1^high^ subpopulation as a potential bridge linking cellular senescence with immune dysregulation in pSjD progression.

Despite these findings, this study has several limitations. First, the precise chemokine signaling pathways mediating CD4⁺ T cell recruitment by DNAJB1^high^ fibroblasts remain to be fully elucidated. Second, the broad-spectrum systemic effects of the D&Q preclude attributing the therapeutic benefits exclusively to senescent fibroblast clearance, highlighting the need to screen for DNAJB1-specific agents. Third, the lack of fibroblast-specific genetic models restricts our ability to establish a definitive in vivo causality between fibroblast senescence and pSjD progression. Fourth, missing age metadata in the public scRNA-seq dataset prevented strictly age-matched bioinformatic analyses, though this was mitigated by rigorous age- or passage-matching in all subsequent experimental validations. Fifth, the limited scale of our clinical validation necessitates larger patient cohorts to corroborate the translational relevance of the JUND-DNAJB1-p21 axis. Finally, computational approaches are inherently predictive and cannot substitute for direct biochemical validation. Accordingly, the therapeutic implications of our findings should be interpreted with these constraints in mind, and future investigations are warranted to address these unresolved questions.

## Conclusions

In conclusion, we identified a distinct DNAJB1^high^ SGF subpopulation that plays an important role in the pathogenesis of pSjD. Our research also provided insights into the mechanism by which IFN-γ promotes DNAJB1 transcription, implicating JUND as a relevant transcriptional regulator that binds to the DNAJB1 promoter under IFN-γ stimulation. Together, these findings suggest that DNAJB1-targeted anti-senescence strategies may serve as a potential therapeutic approach to ameliorate glandular dysfunction in pSjD.

## Supplementary Information

Supplementary Material 1.

Supplementary Material 2.

## Data Availability

The datasets used and/or analyzed during the current study are available from the corresponding author on reasonable request.

## Abbreviations

ACR/EULAR: American College of Rheumatology/European League Against Rheumatism
BEAM: Branch expression analysis modeling
Co-IP: Co-immunoprecipitation
D&Q: Dasatinib and quercetin
DEGs: Differentially expressed genes
FS: Focus score
GO: Gene Ontology
GRN: Gene regulatory network
HC: Healthy control
Hsp: Heat shock protein
HSR: Heat shock response
IACUC: Institutional Animal Care and Use Committee
ICR: Institute of Cancer Research
IFN-γ: Interferon-gamma
IHC: Immunohistochemistry
JBS: JUND-binding site
mIF: Multiplex immunofluorescence
mTOR: Mammalian target of rapamycin
NOD: Non-obese diabetic
PAGA: Partition-based Graph Abstraction
pSjD: Primary Sjögren’s disease
ROC: Receiver operating characteristic
ROS: Reactive oxygen species
SA-β-gal: Senescence-associated β-galactosidase
SASP: Senescence-associated secretory phenotype
SCENIC: Single-Cell rEgulatory Network Inference and Clustering
scRNA-seq: Single-cell RNA sequencing
SGF: Salivary gland fibroblast
SnC: Senescent cell
TF: Transcription factor
TLS: Tertiary lymphoid structure
TSS: Transcription start site
