# Supplementary material for: JUND-DNAJB1-p21 axis drives fibroblast senescence to recruit CD4⁺ T cells in salivary gland pathogenesis of Sjögren’s disease
Source: Immun Ageing. 2026 Aug 11;23:44. doi: 10.1186/s12979-026-00590-8 (PMC13746572; doi:10.1186/s12979-026-00590-8)
Supplement: Supplementary file 1 [file 12979_2026_590_MOESM1_ESM.docx]

**JUND-DNAJB1-p21 axis drives fibroblast senescence to recruit CD4⁺ T cells in salivary gland pathogenesis of Sjögren’s Disease**

Yingliang Shi^1†^, Yi Guo^1†^, Hanlin Tu^2†^, Jiaqi Yue^1^, Zhongyang Zou^1^, Yuyan He^1^, Jing Zhou^1^, Xiang Li^3,4^, Sangang He^1,5*^, Guoliang Sa^1,5*^

^1^ State Key Laboratory of Oral & Maxillofacial Reconstruction and Regeneration, Key Laboratory of Oral Biomedicine Ministry of Education, Hubei Key Laboratory of Stomatology, School & Hospital of Stomatology, Wuhan University, Wuhan 430079, China.

^2^ The Affiliated Stomatological Hospital of Chongqing Medical University

^3^ Department of Stomatology, Huangshi Central Hospital, Affiliated Hospital of Hubei Polytechnic University, China

^4^ Hubei Key Laboratory of Kidney Disease Pathogenesis and Intervention, China, 435000

^5^ Department of Oral and Maxillofacial surgery, School and Hospital of Stomatology, Wuhan University, Wuhan 430079, China

^†^Contributed equally to this work.

^*^Corresponding authors: Guoliang Sa (guoliang@whu.edu.cn); Sangang He (sangang@whu.edu.cn)

**Supplementary** **methods**

**Single-cell RNA sequencing analysis**

Detailed data information can be found in Supplementary Table 1. For downstream analysis, quality control filtering was first performed. The filtering criteria for normal minor salivary gland tissues were nFeature_RNA > 200 & nFeature_RNA < 7500, while those for pSjD minor salivary gland tissues were nFeature_RNA > 200 & nFeature_RNA < 6000 & percent.mt < 30 & nCount_RNA < 15000. After filtering, we processed the data using the Seurat R package (version 4.4.0) following standard workflows, including normalization, highly variable gene identification, scaling, and principal component analysis (PCA) [1]. Batch effects were corrected using the harmony R package (version 1.2.0) for sample integration [2]. Cell clustering was achieved through nonlinear dimensionality reduction implemented with the “FindNeighbors”, “FindClusters”, and “RunUMAP” functions. Cell annotation was performed based on known marker genes, and the annotated data were used for subsequent analyses.

To perform secondary clustering of fibroblasts, we extracted the “Fibroblasts” cluster and repeated the previous steps, with a clustering resolution of 0.5. For visualization, UMAP plots were recalculated using the “RunUMAP” function.

**Senescence score analysis**

To evaluate senescence levels across cell types, we employed the SenMayo gene set for senescence scoring. Compared to other established senescence-related gene sets, SenMayo demonstrates superior fidelity in identifying senescent cells across diverse tissues and species, and has been validated in multiple cell lines and sequencing platforms, including scRNA-seq and bulk RNA-seq [3]. For senescence scoring, we applied the "AddModuleScore", which calculates the module score by comparing the average expression of signature genes against control genes. This approach is widely used for functional module analysis, cell state assessment, and pathway activity quantification. To implement the "AddModuleScore" method and enhance visualization, we utilized the irGSEA R package (version 3.3.1), which incorporates this scoring algorithm [4]. The UMAP density plots of scoring results were generated using the irGSEA R package, while violin plots were created with the ggplot2 R package (version 3.5.1) [5] and the gghalves R package (version 0.1.4), and statistical significance was assessed through the ggpubr R package (version 0.6.0) [6].

In addition, to validate the reliability of the senescence score, we performed the same comparative analysis using four other commonly used scoring methods (including AUCell [7], UCell [8], Singscore [9], and ssGSEA [10]) and four other independent senescent gene sets or pathway (including GenAge [11], Aging Atlas [12], CSGene [13], and KEGG [14]). All scoring methods were implemented using the irGSEA R package (version 3.3.1).

**Identification of differentially expressed genes (DEGs)**

To identify differentially expressed genes (DEGs) in each cell subpopulation, we performed differential expression analysis using the FindAllMarkers() function with parameters set as "only.pos = TRUE, min.pct = 0.25, logfc.threshold = 0.25", while excluding mitochondrial and ribosomal genes. Genes were considered statistically significant when meeting the thresholds of |avg_logFC| > 0.25 and adjusted p-value (p_val_adj) < 0.05. DEGs were visualized using the scRNAtoolVis R package (version 0.1.0) and ggplot2 R package (version 3.5.1).

**Gene function enrichment analysis**

Gene Ontology (GO) and Kyoto Encyclopedia of Genes and Genomes (KEGG) enrichment analyses were performed on DEGs from target fibroblast subpopulations using the "ClusterProfiler" R package (version 4.10.1) [15-17], while Reactome pathway enrichment analysis was conducted using the "ReactomePA" R package (version 1.46.0) [18]. The enrichment results were visualized using the "ggplot2" R package.

**Metacell analysis**

To mitigate issues arising from data sparsity and enhance the reliability of subsequent correlation analyses, we employed the "SuperCell" R package (version 1.0) to merge highly similar fibroblasts into metacells (k.knn = 20, gamma = 20), with subsequent correlation analyses being performed at the metacell level [19].

**Correlation analysis**

To explore the relationships among DNAJB1, senescence, and IFN-γ signaling pathways within fibroblasts, we recalculated module scores at the metacell level using the AddModuleScore. For the IFN-γ signaling pathway, we utilized the REACTOME_INTERFERON_GAMMA_SIGNALING gene set from MSigDB (version v2025.1.Hs) [20-22]. Subsequently, we computed and visualized the correlation of these scores using the ggscatterstats function (type = "spearman") from the ggstatsplot R package (version 0.12.3) [23].

**Cell-cell communication analysis**

Intercellular communication was assessed through systematic quantification of ligand-receptor interactions between distinct cell clusters. The analysis was performed using the CellChat R package (version 1.6.1), with normalized gene expression profiles and annotated cell type data serving as primary inputs [24].

**Receiver operating characteristic (ROC) curve analysis**

The pROC R package (version 1.18.5) was used to display and analyze ROC curves to examine the diagnostic ability of key genes [25]. The bulk RNA-seq data included in the labial gland analysis were GSE173808 [26] and GSE127952, while the bulk RNA-seq data included in the parotid gland analysis were GSE173808 and GSE40611 [27]. Detailed data information can be found in Supplementary Table 1.

**Western blot analysis**

Total proteins were extracted from fibroblasts or mouse submandibular glands using RIPA buffer (P0013B, Beyotime Biotechnology) supplemented with protease inhibitors (04693159001, Roche) and phosphatase inhibitors (04906837001, Roche). Protein concentration was determined by BCA assay (P0012, Beyotime Biotechnology). Samples were separated by SDS-PAGE and transferred to PVDF membranes. After 15 min blocking with rapid blocking buffer (P0252, Beyotime Biotechnology), membranes were incubated with primary antibodies at 4 °C overnight, followed by secondary antibodies for 1 h at room temperature. Protein bands were visualized with the SuperECL Substrate Detection Kit (PMK003, Bioprimacy) and the Odyssey® Fc imaging system (LI-COR). Band intensities were quantified using Fiji software and normalized to β-actin intensity.

The primary antibodies in this study include anti-DNAJB1 (13174-1-AP, Proteintech), anti-p21 (10355-1-AP, Proteintech), anti-p16 (10883-1-AP, Proteintech), anti-JUND (83134-6-RR, Proteintech), and anti-β-actin (66009-1-Ig, Proteintech). Secondary antibodies include: HRP Goat Anti-Rabbit IgG (H+L) (AS014, ABclonal) and HRP Goat Anti-Mouse IgG (H+L) (AS003, ABclonal).

**Histopathological and immunohistochemical (IHC) examinations**

Human labial gland and mouse submandibular gland samples were fixed in 4% paraformaldehyde (PFA) at 4 °C for 24 hours. Following fixation, the tissues underwent dehydration, paraffin embedding, and sectioning into 5 μm thick slices. Sections were stained with hematoxylin and eosin (H&E) according to standard histopathological procedures. To calculate the focus score (FS), the total number of foci (defined as an aggregate of ≥50 lymphocytes localized periductally or perivascularly) in the specimen was divided by the glandular surface area and then multiplied by 4, yielding the number of foci per 4 mm^2^ [28]. Immunohistochemical staining was performed using an UltraSensitive^TM^ SP IHC kit (KIT-9710, MXB) according to the manufacturer's protocol with primary antibodies against DNAJB1 (13174-1-AP, Proteintech), p21 (10355-1-AP, Proteintech), p16 (10883-1-AP, Proteintech), Ki67 (27309-1-AP, Proteintech), and JUND (83134-6-RR, Proteintech). Tissue sections were developed with DAB substrate (ZLI-9018, ZSGB-Bio) and nuclear staining was performed with hematoxylin. The percentage of positive cells was quantified using Fiji software for semi-quantitative analysis of protein levels.

**Supplementary references**

1.	Butler A, Hoffman P, Smibert P, Papalexi E, Satija R. Integrating single-cell transcriptomic data across different conditions, technologies, and species. Nat Biotechnol. 2018;36:411-20.

2.	Korsunsky I, Millard N, Fan J, Slowikowski K, Zhang F, Wei K, et al. Fast, sensitive and accurate integration of single-cell data with Harmony. Nat Methods. 2019;16:1289-96.

3.	Saul D, Kosinsky R L, Atkinson E J, Doolittle M L, Zhang X, LeBrasseur N K, et al. A new gene set identifies senescent cells and predicts senescence-associated pathways across tissues. Nat Commun. 2022;13:4827.

4.	Fan C, Chen F, Chen Y, Huang L, Wang M, Liu Y, et al. irGSEA: the integration of single-cell rank-based gene set enrichment analysis. Brief Bioinform. 2024;25:

5.	Villanueva R A M, Chen Z J. ggplot2: elegant graphics for data analysis. Taylor & Francis; 2019.

6.	Kassambara A. ggpubr:'ggplot2'based publication ready plots. R package version. 2018;2.

7.	Aibar S, González-Blas C B, Moerman T, Huynh-Thu V A, Imrichova H, Hulselmans G, et al. SCENIC: single-cell regulatory network inference and clustering. Nat Methods. 2017;14:1083-6.

8.	Andreatta M, Carmona S J. UCell: Robust and scalable single-cell gene signature scoring. Comput Struct Biotechnol J. 2021;19:3796-8.

9.	Bhuva D D, Cursons J, Davis M J. Stable gene expression for normalisation and single-sample scoring. Nucleic Acids Res. 2020;48:e113.

10.	Krug K, Mertins P, Zhang B, Hornbeck P, Raju R, Ahmad R, et al. A Curated Resource for Phosphosite-specific Signature Analysis. Mol Cell Proteomics. 2019;18:576-93.

11.	Tacutu R, Thornton D, Johnson E, Budovsky A, Barardo D, Craig T, et al. Human Ageing Genomic Resources: new and updated databases. Nucleic Acids Res. 2018;46:D1083-d90.

12.	Aging Atlas: a multi-omics database for aging biology. Nucleic Acids Res. 2021;49:D825-d30.

13.	Zhao M, Chen L, Qu H. CSGene: a literature-based database for cell senescence genes and its application to identify critical cell aging pathways and associated diseases. Cell Death Dis. 2016;7:e2053.

14.	Kanehisa M, Furumichi M, Sato Y, Kawashima M, Ishiguro-Watanabe M. KEGG for taxonomy-based analysis of pathways and genomes. Nucleic Acids Res. 2023;51:D587-d92.

15.	Yu G, Wang L G, Han Y, He Q Y. clusterProfiler: an R package for comparing biological themes among gene clusters. Omics. 2012;16:284-7.

16.	Wu T, Hu E, Xu S, Chen M, Guo P, Dai Z, et al. clusterProfiler 4.0: A universal enrichment tool for interpreting omics data. Innovation (Camb). 2021;2:100141.

17.	Xu S, Hu E, Cai Y, Xie Z, Luo X, Zhan L, et al. Using clusterProfiler to characterize multiomics data. Nat Protoc. 2024;19:3292-320.

18.	Yu G, He Q Y. ReactomePA: an R/Bioconductor package for reactome pathway analysis and visualization. Mol Biosyst. 2016;12:477-9.

19.	Bilous M, Tran L, Cianciaruso C, Gabriel A, Michel H, Carmona S J, et al. Metacells untangle large and complex single-cell transcriptome networks. BMC Bioinf. 2022;23:336.

20.	Subramanian A, Tamayo P, Mootha V K, Mukherjee S, Ebert B L, Gillette M A, et al. Gene set enrichment analysis: a knowledge-based approach for interpreting genome-wide expression profiles. Proc Natl Acad Sci USA. 2005;102:15545-50.

21.	Liberzon A, Subramanian A, Pinchback R, Thorvaldsdóttir H, Tamayo P, Mesirov J P. Molecular signatures database (MSigDB) 3.0. Bioinformatics. 2011;27:1739-40.

22.	Liberzon A, Birger C, Thorvaldsdóttir H, Ghandi M, Mesirov J P, Tamayo P. The Molecular Signatures Database (MSigDB) hallmark gene set collection. Cell Syst. 2015;1:417-25.

23.	Patil I. Visualizations with statistical details: The'ggstatsplot'approach. Journal of Open Source Software. 2021;6:

24.	Jin S, Guerrero-Juarez C F, Zhang L, Chang I, Ramos R, Kuan C H, et al. Inference and analysis of cell-cell communication using CellChat. Nat Commun. 2021;12:1088.

25.	Robin X, Turck N, Hainard A, Tiberti N, Lisacek F, Sanchez J C, et al. pROC: an open-source package for R and S+ to analyze and compare ROC curves. BMC Bioinf. 2011;12:77.

26.	Verstappen G M, Gao L, Pringle S, Haacke E A, van der Vegt B, Liefers S C, et al. The Transcriptome of Paired Major and Minor Salivary Gland Tissue in Patients With Primary Sjögren's Syndrome. Front Immunol. 2021;12:681941.

27.	Horvath S, Nazmul-Hossain A N, Pollard R P, Kroese F G, Vissink A, Kallenberg C G, et al. Systems analysis of primary Sjögren's syndrome pathogenesis in salivary glands identifies shared pathways in human and a mouse model. Arthritis Res Ther. 2012;14:R238.

28.	Fisher B A, Jonsson R, Daniels T, Bombardieri M, Brown R M, Morgan P, et al. Standardisation of labial salivary gland histopathology in clinical trials in primary Sjögren's syndrome. Ann Rheum Dis. 2017;76:1161-8.

**Supplementary Figure**

**Supplementary Fig. 1** Cell type-specific gene expression. Dot plot showing the expression abundance of genes specific to each cell type. The size of the dot represents the percentage of cells expressing the gene, and the color intensity represents the average expression level.

**Supplementary Fig. 2** Cross-validation of fibroblast senescence in pSjD using different gene sets and scoring methods. **A-E** Senescence scores of salivary gland single cells calculated using five different senescence gene sets: SenMayo (A), GenAge (B), AgingAtlas (C), CSGene (D), and KEGG (E). The scores were generated using five different scoring methods: AddModuleScore, AUCell, UCell, Singscore, and ssGSEA. The upper row in each panel shows UMAP density plots, where cells are colored by their senescence score. Yellow indicates the highest gene expression, while dark blue indicates low or no gene expression. The lower row in each panel shows violin plots comparing the senescence scores of fibroblasts from HC (blue) and pSjD (red) groups. Data are presented as mean ± SEM. * p < 0.05, ** p < 0.01, *** p < 0.001, **** p < 0.0001. Unpaired, 2-tailed Student’s t test was used in A-E for statistical analysis.

**Supplementary Fig. 3** Comparison of time and peak memory usage for different scoring methods. **A** Bar chart showing the time elapsed (in seconds) for five different scoring methods: AddModuleScore, AUCell, UCell, Singscore, and ssGSEA. **B** Bar chart showing the peak RAM usage (in megabytes) for the five scoring methods. Data are presented as mean ± SEM. * p < 0.05, ** p < 0.01, *** p < 0.001, **** p < 0.0001. n=10 per group. Mann-Whitney U test was used in A and B for statistical analysis.

**Supplementary Fig. 4** Co-localization of PDGFRα with p21 and Ki-67 in murine submandibular glands. Representative multiplex immunofluorescence (mIF) images demonstrating the expression of the fibroblast marker PDGFRα, p21, and Ki-67 in the submandibular glands. Nuclei were counterstained with DAPI. Scale bars are 50 μm.

**Supplementary Fig. 5** Changes in the proportion of fibroblast subpopulations in pSjD. Dot plots showing the proportion of each fibroblast subpopulation in the HC group (blue dots) and pSjD group (red dots). Each dot represents a biological sample. The horizontal black lines represent the mean value for each group. The p-values are shown for each comparison. Unpaired, 2-tailed Student’s t test was used for statistical analysis.

**Supplementary Fig. 6** Cell-cell communication analysis of IFN-II signaling network. A heatmap showing the communication probability of the IFN-II signaling network. The x-axis represents the receiver cell types, and the y-axis represents the sender cell types.

**Supplementary Fig. 7** Co-localization of PDGFRα, DNAJB1, and p21 in human labial glands. Representative mIF staining images of p21^+^ DNAJB1^+^ fibroblasts in the labial glands of HC and pSjD groups. The white arrows indicate the co-localization of p21, DNAJB1, and PDGFRα. Scale bars are 10 μm and 40 μm.

**Supplementary Fig. 8** Characterization of primary SGFs. Representative immunofluorescence staining images performing to validate SGF identity. Scale bars are 50 µm.

**Supplementary Fig. 9** Identification and compositional analysis of T cell subpopulations. **A** UMAP plot depicting cells colored by T cell subpopulations. **B** Feature plots showing the expression levels of canonical T cell marker genes. CD3D, CD3E, and CD3G are used as pan-T cell markers; CD4 identifies the CD4^+^ T cells, while CD8A and CD8B identify the CD8^+^ T cells subpopulation. The color intensity represents the normalized expression level. **C.** Stacked bar plot showing the relative proportions of CD4^+^ and CD8^+^ T cells in the HC group and pSjD group. The exact percentages for each cell type are indicated within the bars.

**Supplementary Fig. 10** Spatial distribution of DNAJB1^+^ fibroblasts and CD4⁺ T cells. Representative mIF staining images showing DNAJB1^+^ fibroblasts and CD4^+^ T cells in labial glands. Scale bars are 20 μm.

**Supplementary Fig. 11** JUND positively correlates with DNAJB1 expression and directly binds to the DNAJB1 promoter region. **A** Scatter plots showing the correlation between JUND score and IFN-γ score, DNAJB1 score, and senescence score. The Spearman's correlation coefficient (ρ) and p-value are indicated. **B** Schematic of the putative JUND-binding site (JBS) identified in the promoter region of the DNAJB1 TSS, showing the specific sequence and the consensus binding motif logo. **C** AlphaFold-predicted structure of the JUND protein in complex with JBS within the DNAJB1 promoter. The zoomed-in view depicts the protein-DNA interface. Model quality is indicated by ipTM/pTM scores and a pLDDT color scale. The protein structure is colored according to pLDDT as shown in the color scale.

**Supplementary Fig. 12** Western blot analysis and quantification showing the expression of p21 in SGFs treated with different concentrations of D&Q.

**Supplementary Fig. 13** Co-localization of PDGFRα with DNAJB1 and JUND in murine submandibular glands. Representative mIF images demonstrating the expression of the fibroblast marker PDGFRα, DNAJB, and JUND in the submandibular glands. Nuclei were counterstained with DAPI. Scale bars are 50 μm.

| Source | Tissue | Type | Link |
| --- | --- | --- | --- |
| COVID-19 Cell Atlas | labial gland | scRNA-seq | https://www.covid19cellatlas.org/index.healthy.html |
| GSE272409 | labial gland | scRNA-seq | https://www.ncbi.nlm.nih.gov/geo/query/acc.cgi?acc=GSE272409 |
| GSE173808 | labial gland & parotid gland | bulkRNA-seq | https://www.ncbi.nlm.nih.gov/geo/query/acc.cgi?acc=GSE173808 |
| GSE127952 | labial gland | bulkRNA-seq | https://www.ncbi.nlm.nih.gov/geo/query/acc.cgi?acc=GSE127952 |
| GSE40611 | parotid gland | bulkRNA-seq | https://www.ncbi.nlm.nih.gov/geo/query/acc.cgi?acc=GSE40611 |

**Supplementary Table 1.** All sequencing data used in this study.

| **Characteristic** | **Healthy controls**  **(n = 5)** | **pSjD patients**  **(n = 5)** | **P value** |
| --- | --- | --- | --- |
| Age, years (mean ± SD) | 45.6 ± 19.9 | 47.4 ± 11.5 | 0.8650 |
| Female, n (%) | 4 (80.0%) | 4 (80.0%) | 1.0000 |
| Labial gland focus score (foci/4 mm²) | 0 | 2.30 ± 0.79 | 0.0002 |

**Supplementary Table 2.** Clinical and demographic characteristics of healthy controls and pSjD patients.
