# Supplementary material for: JUND-DNAJB1-p21 axis drives fibroblast senescence to recruit CD4⁺ T cells in salivary gland pathogenesis of Sjögren’s disease
Source: Immun Ageing. 2026 Aug 11;23:44. doi: 10.1186/s12979-026-00590-8 (PMC13746572; doi:10.1186/s12979-026-00590-8)
Supplement: Supplementary file 2 [file 12979_2026_590_MOESM2_ESM.docx]

Full uncropped blots for Figure S12
